# The Role of Mitochondrial Homeostasis in the Pathogenesis of Chronic Rhinosinusitis

**DOI:** 10.1002/iid3.70579

**Published:** 2026-11-05

**Authors:** Babak Ghalehbaghi, Morteza Fallahpour, Mohammad Nabavi, Maryam Jalessi, Hadi Ghanbari, Nasibeh Khayer, Farzad Taghizadeh‐Hesary

**Affiliations:** ^1^ ENT and Head and Neck Research Center and Department, School of Medicine, The Five Senses Health Institute Iran University of Medical Sciences Tehran Iran; ^2^ Department of Allergy and Clinical Immunology, Rasool‐e‐Akram Hospital Iran University of Medical Sciences Tehran Iran; ^3^ Skull Base Research Center, The Five Senses Health Institute, School of Medicine Iran University of Medical Sciences Tehran Iran; ^4^ Breast Cancer Research Center Iran University of Medical Sciences Tehran Iran; ^5^ Micro/Nano Technology Center Tokai University Hiratsuka‐shi Kanagawa Japan

**Keywords:** chronic rhinosinusitis, inflammation, mitochondria, nasal polyp, reactive oxygen species

## Abstract

**Background:**

Chronic rhinosinusitis (CRS) is a common clinical condition characterized by persistent inflammation of the nasal and sinus mucosa. While inflammatory endotypes are well‐described, the upstream metabolic dysregulations that initiate and perpetuate inflammation remain poorly defined. This review examines how mitochondrial metabolism, biogenesis, and dysfunction contribute to CRS pathogenesis.

**Methods:**

A comprehensive literature search was performed up to November 2025 using PubMed/MEDLINE, Web of Science, and ScienceDirect. Search terms included “chronic rhinosinusitis,” “mitochondria,” “oxidative stress,” “reactive oxygen species,” “inflammation,” and “epithelial barrier,” together with related keywords and synonyms. Experimental, mechanistic, and translational studies exploring mitochondrial involvement in CRS were included.

**Results:**

Evidence from multiple experimental studies demonstrates that mitochondrial dysfunction is closely intertwined with key pathological processes in CRS. Mitochondria are damaged by diverse CRS‐related pathogenic factors leading to excessive reactive oxygen species production, impaired biogenesis, and disrupted membrane potential. These mitochondrial abnormalities amplify epithelial inflammation, oxidative stress, and barrier dysfunction, creating a self‐propagating vicious cycle in which mitochondrial injury and inflammation reinforce each other. Mitochondrial dysfunction also contributes to characteristic histopathological changes, including epithelial damage, ciliary impairment, and fibrin deposition.

**Conclusions:**

Mitochondria play a pivotal role in CRS pathogenesis. Targeting this self‐amplifying cycle of Inflammation–oxidative stress–mitochondrial dysfunction therefore represents a promising therapeutic strategy for breaking the chronicity of CRS.

## Graphical abstract

The vicious cycle between pathogenic factors‐induced inflammation, oxidative stress, and mitochondrial dysfunction in the context of chronic rhinosinusitis. Created in BioRender. Taghizadeh‐Hesary (2026) https://BioRender.com/k5f4t1f.

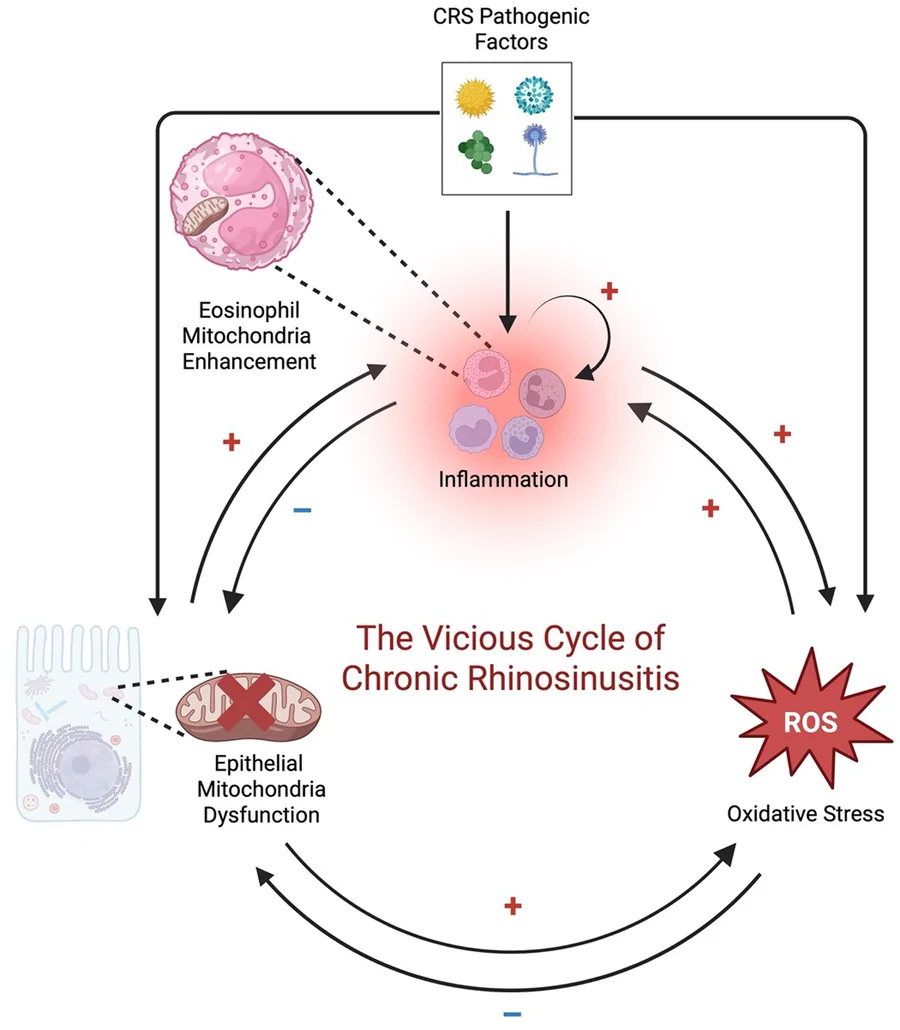

## 1 Introduction

Chronic rhinosinusitis (CRS) is one of the most common chronic inflammatory disorders, affecting approximately 8% of the global population and substantially impairing quality of life [1, 2]. Its prevalence continues to rise, with an estimated 19% increase reported between 2014 and 2020 [1]. CRS is a heterogeneous condition defined by persistent inflammation of the upper airway mucosa lasting at least 12 weeks [3].

Experimental and clinical studies have described three major inflammatory endotypes (T1, T2, and T3), each characterized by distinct cytokine profiles. Historically, CRS has been classified according to the presence or absence of nasal polyps into CRS with nasal polyps (CRSwNP) and CRS without nasal polyps (CRSsNP). However, growing evidence indicates that both phenotypes exhibit considerable inflammatory heterogeneity, and neither can be reliably categorized by inflammatory type alone [4]. The sinonasal epithelium plays a central role in disease pathogenesis. In CRS, epithelial cells display impaired regulatory capacity and fail to appropriately downregulate inflammatory signaling, contributing to chronic and self‐sustaining mucosal inflammation [4, 5, 6]. Despite extensive research, the underlying pathophysiology of CRS remains incompletely understood, in part due to its substantial clinical and molecular heterogeneity [4].

Mitochondria are intracellular organelles with diverse applications and benefits for human cells. They have been largely considered as the cellular energy source. However, more recent evidence has revealed their crucial importance in different human diseases, including neurodegeneration, cardiovascular diseases, metabolic syndrome, and cancer [7]. Mitochondria, once viewed primarily as cellular energy producers, are now recognized as key regulators of cellular homeostasis, innate immunity, oxidative stress responses, and inflammatory signaling [8]. Emerging evidence suggests that mitochondrial metabolism is disrupted in CRS, particularly in CRSwNP. Using machine‐learning approaches, Yan and colleagues identified five mitochondrial‐related genes, including ALDH1L1, BCKDHB, CBR3, HMGCS2, and OXR1, that were consistently downregulated in nasal epithelial cells from CRSwNP patients [9]. Similarly, Yoon and colleagues demonstrated that *Staphylococcus aureus* enterotoxin B (SEB), a major contributor to CRS pathogenesis, alters mitochondrial morphology, dynamics, and respiratory function in CRSwNP‐derived epithelial cells [10]. Importantly, human specimen studies provide direct evidence of mitochondrial dysfunction. In nasal polyps and aged nasal mucosa, transmission electron microscopy revealed structural mitochondrial abnormalities (reduced size, floating mitochondria, vacuoles), accompanied by reduced manganese superoxide dismutase (SOD2) and increased DRP‐1 expression in submucosal glands [11]. A complementary study confirmed increased endoplasmic reticulum (ER)‐mitochondria tethering, elevated cleaved caspase‐3, and upregulated DRP‐1/Mfn‐1 in nasal polyps [12]. Together, these human tissue‐based findings directly support mitochondrial dysfunction in CRS pathogenesis.

Given these findings, this review synthesizes current evidence on CRS pathogenesis through the lens of mitochondrial metabolism and biogenesis, highlighting how mitochondrial dysfunction may contribute to the initiation and perpetuation of CRS. This article provides a multifaceted review of CRS pathogenesis, outlining how epithelial mitochondrial function is altered by recognized CRS‐related pathogenic factors, how mitochondrial dysfunction contributes to the self‐perpetuating cycle of chronic inflammation, and how these mitochondrial abnormalities are reflected in the characteristic histopathological changes of the sinonasal epithelium.

## 2 Methods

Relevant literature was systematically searched in PubMed/MEDLINE, Web of Science, and ScienceDirect up to November 2025, limited to English‐language publications. Search keywords included “chronic rhinosinusitis,” “mitochondria,” “oxidative stress,” “reactive oxygen species,” “inflammation,” and “epithelial barrier,” along with related synonyms. Eligible publications included original research articles, experimental and clinical studies, and reviews that examined the mechanistic, pathogenic, diagnostic, or regulatory roles of mitochondria in CRS. Studies unrelated to mitochondrial biology were excluded. The reference lists of selected articles were also manually screened to identify additional relevant studies and ensure comprehensive coverage.

## 3 CRS Pathophysiology

### 3.1 Current Knowledge on CRS Pathogenesis

CRS is a multifactorial and heterogeneous inflammatory condition of the sinonasal mucosa, arising from persistent immune dysregulation at the interface of host epithelium and environmental exposures. This immunological response usually arises from exposures to various environmental stimuli, including allergens, bacteria, viruses, and fungi. Recent advances in immunologic profiling have led to the classification of CRS into distinct endotypes based on the dominant type of inflammatory response: Type 1 (T1), characterized by interferon‐γ (IFN‐γ)‐driven inflammation and neutrophilic infiltration; Type 2 (T2), dominated by IL‐4, IL‐5, and IL‐13 with prominent eosinophilia; and Type 3 (T3), associated with IL‐17 and neutrophilic inflammation (refer to Section 3.2). Both CRSwNP and CRSsNP clinical phenotypes can exhibit each of these endotypes. However, many patients demonstrate a mixed endotype or even lack a clear elevation of T1, T2, or T3 markers, underscoring the complex and variable nature of CRS pathogenesis [4]. Importantly, epithelial cells are the primary targets of immune‐dysregulation in CRS, rendering them central to the initiation and perpetuation of chronic inflammation [5]. Physiologically, epithelial cells are equipped with a range of intrinsic defense mechanisms that facilitate an efficient return to homeostasis in healthy individuals. However, in the context of CRS, these protective responses are often impaired, leading to a failure in resolving the self‐perpetuating inflammatory process [13]. These defense strategies include (Figure 1):

**Figure 1:**
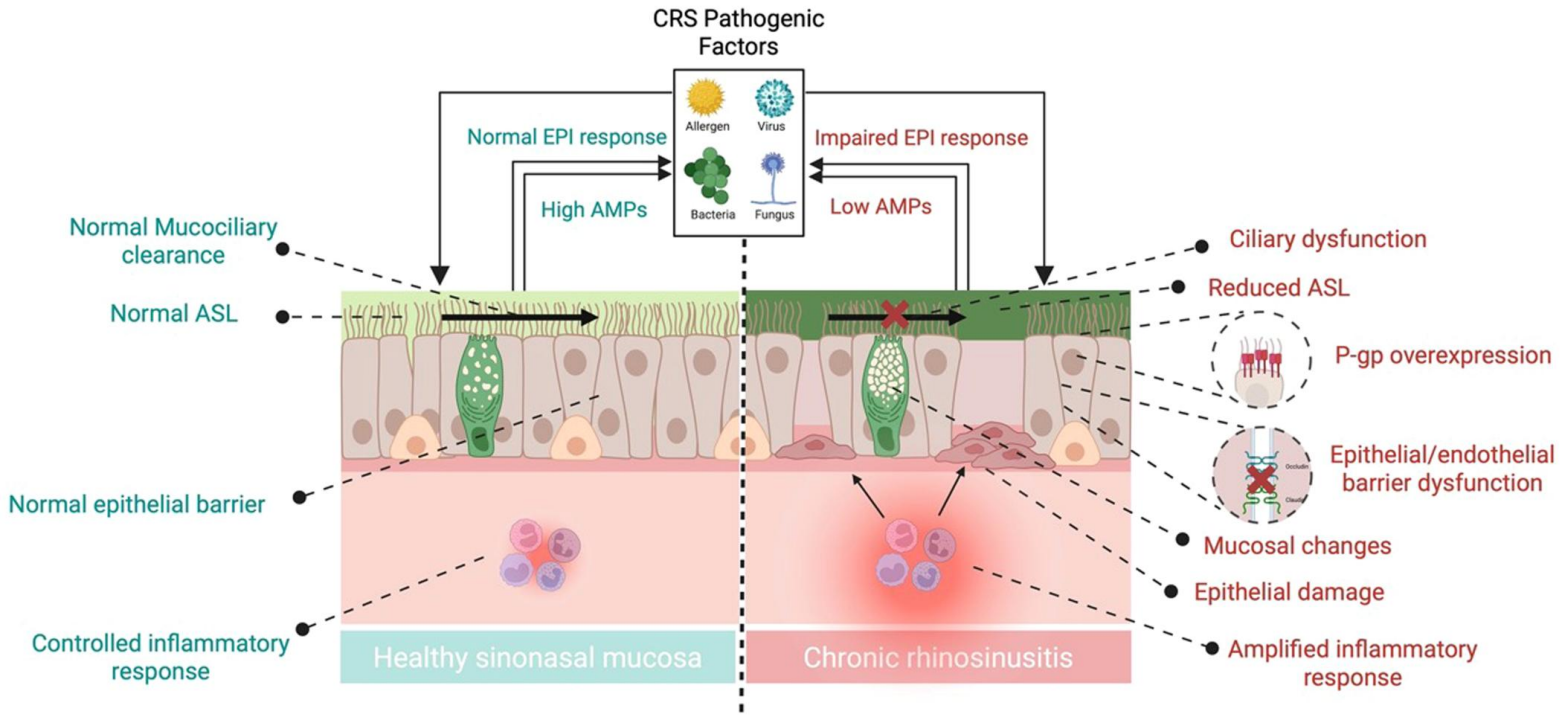
Schematic representation of epithelial barrier function in health and chronic rhinosinusitis (CRS). In healthy individuals, epithelial cells maintain mucosal homeostasis via intact mucociliary clearance, robust antimicrobial peptide (AMP) production, and endogenous protease inhibitor (EPI) responses. In CRS, environmental and microbial triggers impair these defenses, leading to epithelial barrier dysfunction, reduced airway surface liquid (ASL), P‐glycoprotein (P‐gp) overexpression, ciliary dysmotility, and amplified inflammatory responses. These changes drive persistent inflammation and tissue remodeling, contributing to disease chronicity. AMPs, antimicrobial peptides; ASL, airway surface liquid; EPIs, endogenous protease inhibitors; P‐gp, P‐glycoprotein. Created in BioRender. Taghizadeh‐Hesary (2026) https://BioRender.com/03dfofl.

(1) The mucociliary clearance (MCC) system, essential for removing inhaled pathogens and debris, can be disrupted by various microbial pathogens, including *Haemophilus influenzae*, *Streptococcus pneumoniae*, *S. aureus*, *Aspergillus fumigatus*, and *Pseudomonas aeruginosa* [14]. Additionally, genetic conditions such as cystic fibrosis (CF) and primary ciliary dyskinesia enhance the risk of CRS by impairing the clearance mechanism [15]; (2) antimicrobial peptides (AMPs) such as S100A7 (psoriasin) and S100A8/9 (calprotectin) are critical components of epithelial defense. Studies have demonstrated a marked reduction in the AMPs in the context of CRSwNP, rendering the epithelium more susceptible to microbial colonization and immune activation [6]; (3) another key factor is the airway surface liquid (ASL), which facilitates MCC. In CRSwNP, ASL volume is frequently diminished due to downregulation of PLUNC family proteins (specifically SPLUNC1) and upregulation of the anion exchanger Pendrin/SLC26A4. The latter promotes excessive anion exchange, contributing to ASL depletion and impaired mucociliary function [16, 17, 18]; (4) the epithelial cell also expresses P‐glycoprotein (P‐gp), a membrane transporter that regulates inflammatory responses to external triggers. Elevated P‐gp levels have been observed in CRSwNP, suggesting a compensatory or maladaptive response to chronic stimulation [19]; and (5) endogenous protease inhibitors (EPIs) represent another layer of epithelial defense. Protease inhibitors such as cystatin A, SPINK5, and cystatin SN function to neutralize exogenous proteases under physiological conditions. However, their expression patterns in CRSwNP vary. Cystatin A and SPINK5 expressions are downregulated in CRS [20]. On the other hand, cystatin SN expression is upregulated in eosinophilic (T2) CRSwNP [21]. It has been demonstrated that cystatin SN overexpression can paradoxically enhance thymic stromal lymphopoietin (TSLP)‐mediated immune responses and promote eosinophil recruitment by releasing IL‐5 and through activating periostin release from fibroblasts [22, 23, 24].

Notably, external triggers may also independently compromise the epithelial barrier. Microbial factors, such as those secreted by *P. aeruginosa* and *S. aureus*, disrupt tight junctional proteins, including occludin and claudin‐1, weakening epithelial integrity [25, 26]. Furthermore, immune cells can aggravate epithelial damage via cytokine‐mediated pathways: IL‐4/IL‐13 in T2 and IFN‐γ/granzymes in T1. It has been demonstrated that the Janus kinase/signal transducer and activator of transcription (JAK/STAT) signaling pathway plays a crucial role in cytokine‐induced epithelial alterations in CRSwNP. The stimulation of epithelial cells with IL‐13 and IFN‐γ can activate JAK‐mediated phosphorylation of STAT nuclear transcription factors, leading to transcriptional regulation of genes involved in CRSwNP pathogenesis. These changes include abnormal ciliary differentiation, disruption of tight junctions, increased mucus production, and enhanced release of inflammatory mediators. Zhuang and colleagues demonstrated that ruxolitinib, a selective inhibitor of JAK1/2, could effectively reduce the severity of CRSwNP burden in a rat model with reversing the aforementioned CRS‐related epithelial changes [27].

Disruption of epithelial integrity can increase the exposure to environmental antigens and pathogens, further boosting the inflammatory cascade [4]. The epithelial barrier, in turn, releases epithelial *alarmins*, including IL‐33, IL‐25, TSLP, and B‐cell activating factor, enhancing immune activation and perpetuating the inflammatory cycle [28]. Adding another layer of complexity, pathogen‐derived proteases can activate epithelial cytokine production by stimulating the release of EPIs like cystatin SN. Although this represents a physiological countermeasure, cystatin SN has been shown to upregulate TSLP expression in the epithelium, thereby exacerbating T2‐type inflammation [23]. Moreover, cystatin SN can activate local fibroblasts to secrete periostin, which in turn activates eosinophils, further contributing to T2‐mediated pathology [24]. In addition, staphylococcal alpha‐toxin and *P. aeruginosa* lipopolysaccharide (LPS) can disrupt the endothelial tight junctions by activating sphingomyelinase [29, 30]. The resulting endothelial barrier dysfunction may lead to plasma leakage, activation of the coagulation cascade, and intramucosal fibrin deposition, all of which contribute to nasal polyp formation [4].

In a nutshell, the pathophysiology of CRS can be envisioned as a maladaptive immunological response to external triggers. In susceptible population, this dysregulation propagates through successive immune layers, culminating in epithelial barrier disruption, sustained inflammation, and the formation of nasal polyps. In the setting of CRS, there is a failure to downregulate the inflammatory cascade, leading to a persistent and self‐perpetuating immune response [10]. This conceptual framework highlights the intricate interplay between immune dysregulation and epithelial dysfunction, underscoring potential targets for therapeutic intervention and further elucidation of disease mechanisms.

### 3.2 The Immunopathological Fortress: A Conceptual Framework for CRS

This section introduces a metaphor for CRS pathogenesis, portraying it as a prolonged and misdirected battle between the immune system and the epithelial barrier as follows (Figure 2):

**Figure 2:**
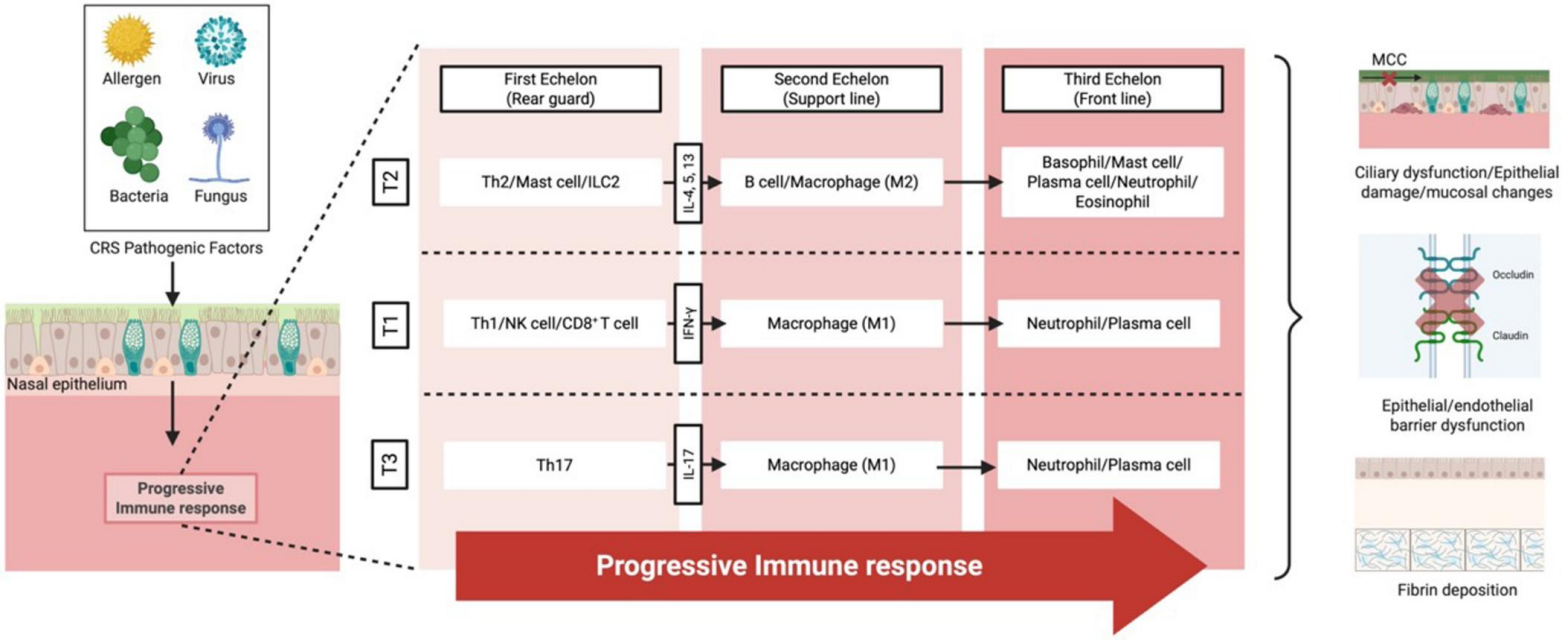
Schematic representation of the progressive immune response in chronic rhinosinusitis. Upon exposure to CRS‐related pathogenic factors such as allergens, viruses, bacteria, or fungi, the nasal epithelium initiates an immune response that escalates across three immunological echelons. The first echelon (rear guard) involves Th2, mast cells, and ILC2 (T2), Th1, NK cells, and CD8^+^ T cells (T1), as well as Th17 cells (T3). These cells release key cytokines that drive recruitment and activation of second‐echelon effector cells, including B cells and macrophages (M1/M2). In the third echelon (front line), plasma cells, neutrophils, eosinophils, basophils, and mast cells execute the inflammatory response. This progressive immune escalation results in epithelial and mucosal damage, including ciliary dysfunction, mucosal changes, tight junction disruption, and fibrin deposition. CD8^+^, cluster of differentiation 8–positive; IFN‐γ, interferon‐gamma; IL‐4, interleukin‐4; IL‐5, interleukin‐5; IL‐13, interleukin‐13; IL‐17, interleukin‐17; ILC2, group 2 innate lymphoid cell; MCC, mucociliary clearance; NK cell, natural killer cell; Th1, T helper 1 cell; Th2, T helper 2 cell; Th17, T helper 17 cell; T1, type 1 immune response; T2, type 2 immune response; T3, type 3 immune response. Created in BioRender. Taghizadeh‐Hesary (2026) https://BioRender.com/kssz9vu.

At the onset of this immune confrontation, various pathogenic factors such as allergens, bacteria, viruses, and fungi initiate the dysregulation of the first echelon of immune cells, referred to here as the “rear guard” of immune defense. This line varies by endotype: in T2 inflammation, group 2 innate lymphoid cells (ILC2), T helper 2 (Th2) cells, and mast cells dominate; in T1, CD8^+^ cytotoxic T cells, natural killer (NK) cells, and Th1 cells are prevalent; while in T3, Th17 cells serve as key players [4]. These first‐echelon cells, once activated, release a cascade of pro‐inflammatory cytokines (IL‐4, IL‐5, and IL‐13 in T2 inflammation; IFN‐γ in T1; and IL‐17 in T3) leading to the recruitment and activation of second echelon, the “support line.” In T2 CRS, this includes M2‐polarized macrophages and B cells; in T1 and T3 types, M1 macrophages take center stage. These support cells further propagate immune responses, amplifying the inflammatory milieu. Subsequently, a tertiary wave of effector immune cells (termed the “front line”) is recruited to directly target the epithelial barrier. In T2 inflammation, basophils, mast cells, plasma cells, neutrophils, and eosinophils infiltrate the tissue. In T1 and T3, plasma cells and neutrophils comprise the primary infiltrates. In this metaphor, the cytokines (IL‐4, IL‐5, IL‐13, IFN‐γ, and IL‐17) serve as biochemical weapons that inflict damage across the mucosal layer.

According to this metaphor, the nasal polyp can be seen as the pathological consequence of a prolonged and unresolved conflict between the immune system and the epithelial barrier, a biological structure akin to a war‐torn stronghold. Nasal polyp would represent a fortress built not for strategic defense, but as a manifestation of immune misdirection and chronic inflammation. In this scenario, the epithelial cells are the defenders or civilians striving to maintain mucosal integrity, while the dysregulated immune cells act as invading forces launching relentless attacks. The resulting tissue alterations, including edema, glandular hyperplasia, and extracellular matrix remodeling, reflect the lasting structural damage driven by this ongoing immunological battle.

## 4 The Role of Mitochondria in Cellular Metabolism

Cellular metabolism encompasses the biochemical reactions that sustain life by converting nutrients into energy and macromolecules, while maintaining cellular integrity and function. It is broadly categorized into: (1) catabolism: breakdown of carbohydrates, lipids, and proteins to generate adenosine triphosphate (ATP, the main cellular energy carrier) and reducing equivalents (reduced form of nicotinamide adenine dinucleotide [NADH] and reduced form of flavin adenine dinucleotide [FADH_2_]); and (2) anabolism: biosynthesis of macromolecules like nucleotides, amino acids, and lipids, essential for cell growth and division [31].

Mitochondria are double‐membrane organelles containing their own circular mitochondrial DNA (mtDNA) and highly folded inner membrane cristae that house the protein complexes essential for oxidative metabolism (Figure 3A). Within epithelial cells, these organelles orchestrate the conversion of nutrients into usable energy through a series of interconnected metabolic pathways. Glucose‐derived pyruvate enters the mitochondrion and is converted into acetyl‐CoA, which feeds into the tricarboxylic acid (TCA) cycle (Figure 3B). As the TCA cycle progresses, intermediates such as citrate, α‐ketoglutarate, succinate, fumarate, and malate are sequentially generated, producing reducing equivalents (NADH and FADH_2_) and a small amount of ATP (Figure 3C). These reducing equivalents are then funneled into the electron transport chain (ETC) embedded in the inner mitochondrial membrane, where complexes I–IV transfer electrons and pump protons into the intermembrane space, establishing an electrochemical gradient. ATP synthase subsequently harnesses this proton motive force to drive the phosphorylation of ADP to ATP, completing oxidative phosphorylation (Figure 3D). Through this coordinated integration of the TCA cycle and ETC, mitochondria serve as the central hub of cellular metabolism, linking nutrient oxidation to efficient ATP production [32].

**Figure 3:**
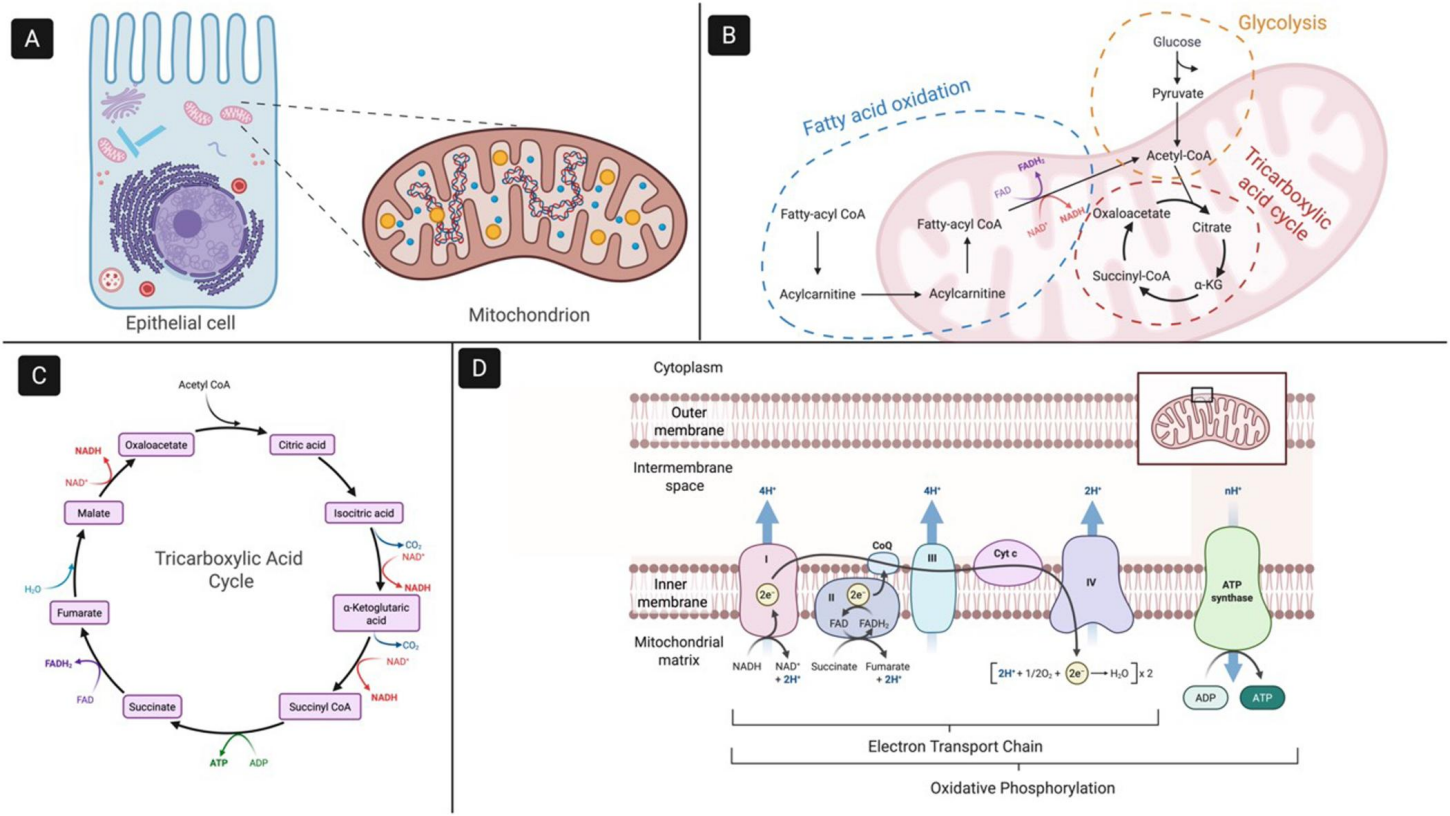
Overview of cellular energy metabolism and mitochondrial function. (A) Schematic representation of a eukaryotic cell highlighting the mitochondrion as the major site of energy production. (B) The major mitochondrial metabolic pathways, showing how glycolysis‐derived pyruvate is converted into acetyl‐CoA to fuel the tricarboxylic acid (TCA) cycle, while fatty acids are transformed into acyl‐CoA and transported into mitochondria as acylcarnitine to undergo fatty‐acid oxidation. (C) The TCA cycle oxidizes acetyl‐CoA to generate reducing equivalents in the form of NADH and FADH_2_, along with ATP. (D) The electron transport chain, located in the inner mitochondrial membrane, utilizes NADH and FADH_2_ to drive proton pumping across the membrane via complexes I–IV, generating a proton gradient used by ATP synthase (complex V) to produce ATP. ADP, adenosine diphosphate; ATP, adenosine triphosphate; CoA, coenzyme A; CoQ, coenzyme Q; Cyt c, cytochrome c; ETC, electron transport chain; FAD, flavin adenine dinucleotide; FADH_2_, reduced flavin adenine dinucleotide; KG (α‐KG), alpha‐ketoglutarate; NAD^+^, oxidized nicotinamide adenine dinucleotide; NADH, reduced nicotinamide adenine dinucleotide. Created in BioRender. Taghizadeh‐Hesary (2026) https://BioRender.com/o4dyxgj.

## 5 CRS Pathogenesis Through the Lens of Mitochondrial Homeostasis

Emerging evidence addresses the importance of mitochondrial homeostasis in the pathogenesis of CRS. This section explores how mitochondrial biology is involved in the pathogenesis of CRS. We begin by examining how various CRS‐related triggers and risk factors disrupt mitochondrial homeostasis within the nasal epithelium. Next, we outline the immune response to these pathogenic factors and the corresponding histopathological changes through the lens of mitochondrial function. Finally, we consider the potential connections between CRS‐related genetic factors and mitochondrial biology.

### 5.1 Epithelial Mitochondrial Damage in Exposure to CRS‐Related Pathogenic Factors

Here we show how different CRS‐related pathogenic factors can interfere with the epithelial cells' mitochondrial homeostasis (Figure 4).

**Figure 4:**
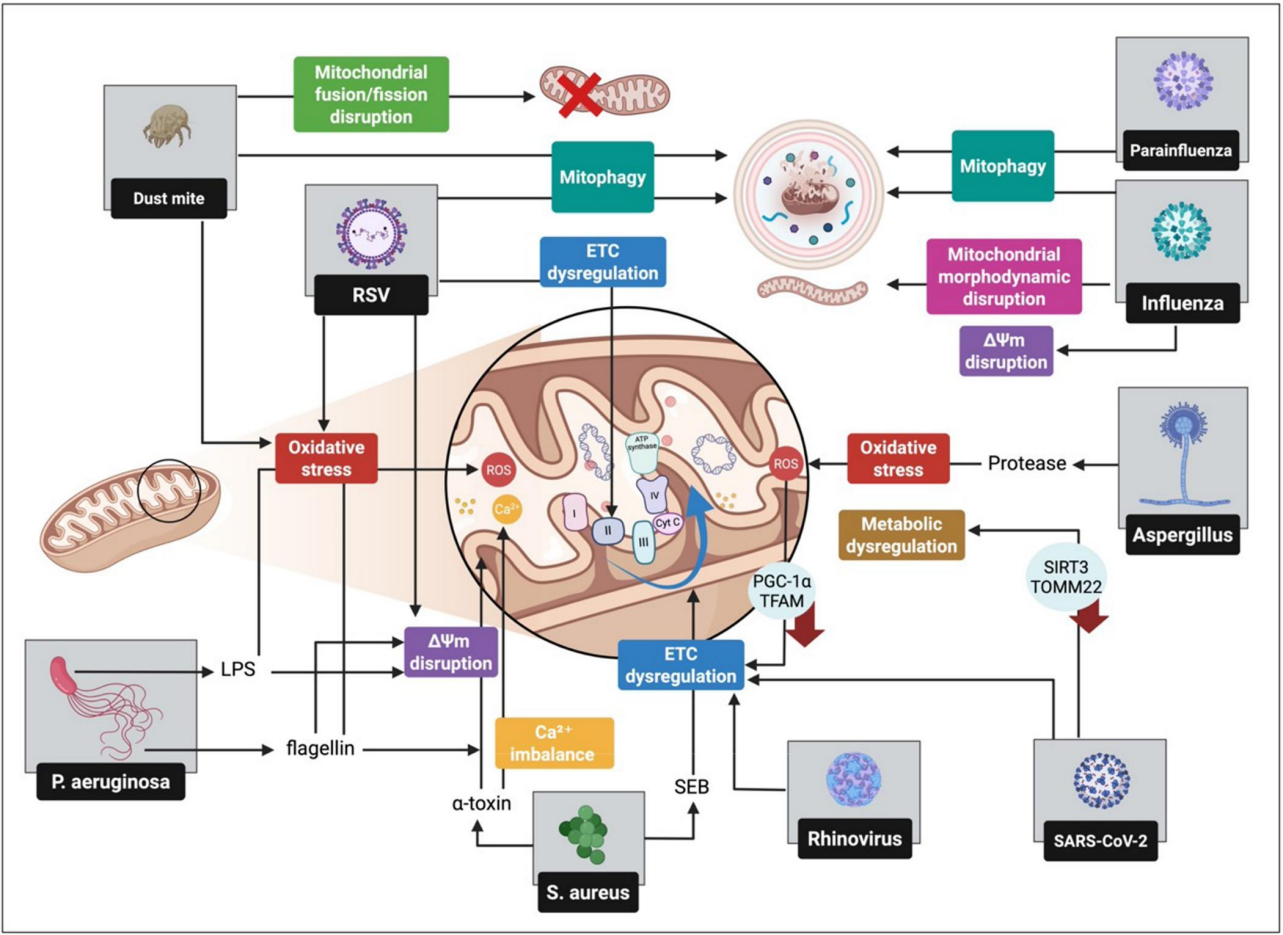
Schematic overview of mitochondrial dysfunctions induced by chronic rhinosinusitis–associated pathogenic factors. Various pathogenic factors (gray boxes), including bacteria, viruses, fungi, and allergens, contribute to mitochondrial dysfunction through distinct but overlapping mechanisms (colored boxes), including oxidative stress, disrupted mitochondrial dynamics (fusion/fission), mitophagy, electron transport chain dysregulation, mitochondrial membrane potential disruption, calcium imbalance, and metabolic dysregulation. ATP, adenosine triphosphate; Cyt c, cytochrome c; ΔΨ_m_, mitochondrial membrane potential; ETC, electron transport chain; LPS, lipopolysaccharide; PGC‐1α, peroxisome proliferator‐activated receptor gamma coactivator 1‐alpha; ROS, reactive oxygen species; SEB, staphylococcal enterotoxin B; SIRT3, sirtuin 3; TFAM, mitochondrial transcription factor A; TOMM22, translocase of outer mitochondrial membrane 22. Created in BioRender. Taghizadeh‐Hesary (2026) https://BioRender.com/6jftphd.

#### 5.1.1 Staphylococcus aureus

*S. aureus* plays a pivotal role in the pathogenesis of CRS by promoting persistent inflammation and disrupting epithelial barrier function [33]. According to a recent systematic review on 57 studies encompassing over 10,000 sinus specimens worldwide, methicillin‐susceptible *S. aureus* constitutes the second most common bacteria in CRS [34]. *S. aureus* can release various enterotoxins that function as superantigens and activate immune responses [35], induce pro‐apoptotic signaling in phagocytes via type I interferon (IFN‐I) pathways in macrophages [36], and contribute to biofilm formation, which shields the bacteria from host defenses and antimicrobial treatments, thereby sustaining chronic inflammation [37]. Its capacity to form biofilms is associated with unfavorable evolution after endoscopic sinus surgery, supporting its clinical relevance in disease persistence [38]. In the following, it is outlined how *S. aureus* can disrupt mitochondrial homeostasis:

(1) *S. aureus* enterotoxin B: *S. aureus* enterotoxins, particularly SEB, have emerged as important pathogenic factors in CRS. Acting as a superantigen, SEB induces intense and sustained mucosal inflammation by nonspecifically activating T cells and promotes the release of type 2 cytokines [35]. Studies have identified significantly higher rates of *S. aureus* SEB positivity among patients with CRSwNP compared to those with CRSsNP and healthy controls, implicating these toxins in nasal polyp pathogenesis [39]. SEB can affect the nasal epithelium by disrupting epithelial tight junctions, inducing ER stress, and activating the immune reaction [35, 40, 41].

Beyond these mechanisms, SEB exerts direct cytotoxic effects on epithelial cells' mitochondria. A pivotal study by Yoon and colleagues explored how SEB impacts mitochondrial morphology, dynamics, and function in nasal epithelial cells derived from CRSwNP patients. This study demonstrated that mitochondria in nasal polyps are elongated and have higher rates of fusion and fission compared with normal mucosal tissue. This study indicated that following SEB exposure, Complex II expression (a component of ETC) was significantly upregulated with low‐dose SEB (0.1 μg/mL), followed by a dose‐dependent decline at higher concentrations. Similarly, Complex III expression increased significantly across low to moderate SEB doses (0.1–5 μg/mL). Complex IV was also induced but only at low concentrations. Critically, Complex V (ATP synthase) activity was inhibited in a dose‐dependent manner, with higher doses of SEB producing more pronounced suppression [10]. This selective activation of upstream OXPHOS Complexes (II, III, IV) in the face of Complex V inhibition leads to a bioenergetic mismatch. The analogy of this condition can be seen with the oligomycin treatment, which is a specific Complex V inhibitor [42]. Specifically, inhibited ATP synthase in the context of active upstream Complexes impedes H^+^ influx into the matrix, resulting in accumulation of H^+^ in the intermembrane space and a consequent hyperpolarization of the mitochondrial membrane (ΔΨ_m_). This backpressure impedes electron flow across the ETC and promotes electron leakage, particularly from Complexes I and III. These leaked electrons react with molecular oxygen to produce superoxide (O_2_^−^), initiating a mitochondrial oxidative burst characterized by excessive mitochondrial reactive oxygen species (mtROS) generation [42]. This mtROS surge leads to: (1) oxidative damage to mtDNA and proteins [43]; (2) triggering of epithelial apoptosis [44]; and (3) disruption of epithelial tight junctions [45], thereby weakening the epithelial barrier.

(2) *S. aureus* alpha‐toxin: *S. aureus* α‐toxin has emerged as a key virulence factor that disrupts epithelial integrity and cellular homeostasis, in part through mitochondrial dysfunction. Early evidence by Yun and colleagues showed that human nasal ciliated epithelial cells exposed to α‐toxin exhibited a biphasic response in ciliary beat frequency, with initial stimulation followed by irreversible decline. This functional loss was paralleled by ultrastructural alterations, notably mitochondrial swelling, suggesting mitochondrial injury as an underlying mechanism [46]. Alpha‐toxin can affect mitochondrial homeostasis through several mechanisms: (1) disruption of mitochondrial calcium homeostasis; Stelzner and colleagues demonstrated that *S. aureus* induces profound disturbances in intracellular calcium signaling. Exposure of airway epithelial cells to α‐toxin‐rich supernatant led to cytosolic Ca^2+^ overload, partly from ER stores and extracellular influx. This calcium surge triggered secondary mitochondrial Ca^2+^ uptake, a known precipitant of mitochondrial permeability transition pore (mPTP) opening, which can lead to mitochondrial swelling [47]. (2) Disruption of mitochondrial membrane potential; Baaske and colleagues found that α‐toxin exposure causes a measurable decline in ΔΨ_m_ and cellular ATP levels. This effect was attributed to both impaired ATP synthesis and pore‐mediated ATP efflux. Importantly, these pores were specific to functional α‐toxin, as mutant forms incapable of pore formation failed to induce ATP release or mitochondrial changes [48]. Collectively, these findings put forward mitochondria as the target of *S. aureus* alpha‐toxin.

#### 5.1.2 Pseudomonas aeruginosa

*P. aeruginosa* may impair epithelial function through multiple mechanisms, including impaired MCC, disruption of epithelial tight junctions, and immune dysregulation [26, 49]. Its ability to form biofilms is linked to poorer outcomes following endoscopic sinus surgery, underscoring its clinical significance in sustaining the disease [38]. Specific clinical conditions, such as Sjogren's syndrome, can increase the risk of *P. aeruginosa* colonization in CRS [50].

Emerging evidence suggests that *P. aeruginosa* may also contribute to CRS by disrupting mitochondrial metabolism in epithelial cells. Rimessi and colleagues demonstrated that *P. aeruginosa* induces mitochondrial dysfunction in airway epithelial cells via a flagellin‐dependent mechanism. Upon infection of CF‐derived IB3‐1 cells, *P. aeruginosa* triggered marked mitochondrial abnormalities, including reduced ΔΨ_m_, mitochondrial fragmentation, swelling, and disrupted cristae architecture. This was accompanied by increased mtROS and impaired Ca^2+^ uptake, culminating in activation of the NLRP3 inflammasome and elevated IL‐1β and IL‐18 release. Crucially, infection with a flagellin‐deficient PAK mutant failed to reproduce these effects, identifying flagellin–TLR5 signaling as the key mediator [51]. These findings reveal that *P. aeruginosa* exploits flagellin–TLR5 signaling to induce mitochondrial Ca^2+^ imbalance and oxidative stress, promoting epithelial inflammation through mitochondrial pathways.

In addition, *P. aeruginosa* LPS would take part in the CRSwNP pathogenesis. Specifically, *P. aeruginosa* LPS may impair endothelial function through mitochondria‐mediated mechanisms. Zhu and colleagues demonstrated that LPS exposure leads to degradation of the endothelial glycocalyx, evidenced by increased shedding of heparan sulfate and upregulation of the glycocalyx‐degrading enzyme heparanase. Crucially, the disruption extended to tight junctions, with decreased expression of occludin and ZO‐1 mediated through increased phosphorylation of myosin light chain, further compromising endothelial barrier function [30]. This study found that mitochondrial metabolism is involved in LPS‐induced endothelial cell barrier dysfunction, characterized by increased mitochondrial swelling, elevated mtROS, and altered ΔΨ_m_ [30]. In the context of CRS, these findings may explain how LPS contributes to nasal polyp formation [52]. As outlined in Section 3.1, the resulting endothelial leakage, followed by fibrin deposition, likely promotes chronic inflammation and tissue remodeling, ultimately leading to the development of edematous nasal polyps [4].

#### 5.1.3 Aspergillus

Accumulating evidence implicates *Aspergillus* species in the pathogenesis of CRS. A cross‐sectional study identified *Aspergillus* as the most prevalent isolated fungus (45%) among CRS patients undergoing surgery [53]. Furthermore, molecular analysis by ITS1/ITS2 PCR identified *Aspergillus* as the predominant genus (79%), with *A. fumigatus* detected in 66% of all samples [54]. *Aspergillus* species may contribute to the development of CRS through multiple mechanisms, including mucociliary dysfunction and the induction of local inflammation [14]. The latter is partly mediated by the upregulation of inflammatory cytokines in sinonasal epithelial cells. Dietz and colleagues demonstrated that *A. fumigatus* can exacerbate nasal mucosal inflammation in CRS by triggering the release of IL‐33 in a protease‐activated receptor 2 (PAR2)‐dependent manner [55].

*Aspergillus* can directly impair mitochondrial metabolism in airway epithelial cells. Kim and colleagues demonstrated that *Aspergillus* proteases enhance the release of mtROS. The resulting mtROS can reduce mitochondrial ATP synthesis by disrupting the ETC and OXPHOS, mediated through downregulation of peroxisome proliferator‐activated receptor gamma coactivator‐1 alpha (PGC‐1α) and mitochondrial transcription factor A (TFAM) expression. Moreover, the study showed that mitochondrial dysfunction leads to the impairment of intercellular junctions by decreasing the expression of ZO‐1, claudin‐1, occludin, and E‐cadherin, as a consequence of mtROS‐induced gene regulation [56].

#### 5.1.4 Viruses

Viral infections are increasingly recognized as important contributors to the pathogenesis of CRS. A recent systematic review on 30 studies concluded that viral prevalence in nasal specimens of CRS subjects was higher compared to controls in most studies, with rhinovirus being the most common virus detected [57]. A 2025 population‐based study found that viral infections lead to a greater than twofold increased risk of developing CRS within 1 year, and recurrent viral infections pose a greater risk than single episodes [58]. Rhinoviruses, respiratory syncytial virus (RSV), influenza, parainfluenza, adenoviruses, and more recently SARS‐CoV‐2, have all been implicated in initiating or exacerbating CRS. A large propensity‐matched study found that CRS diagnosis risk significantly increased following a COVID‐19 infection (relative risk [RR] = 2.13). Risk varied by variant: highest during the Omicron period (RR = 2.10), lowest during the Delta period (RR = 1.20) [59]. Beyond their role in acute upper respiratory illnesses, viruses can disrupt MCC [60], damage the epithelial barrier [61], and trigger persistent inflammatory responses in the sinonasal mucosa [57]. These viruses can both impair local immune defenses and facilitate secondary bacterial colonization [57].

Compelling evidence identifies mitochondria as key targets of various CRS‐related viruses. For instance, rhinovirus has been shown to impair mitochondrial respiration and induce proton leakage [62]; RSV can lead to ΔΨ_m_ loss, impaired mitochondrial respiration, increased mtROS production, and mitophagy activation [63, 64]; influenza virus can disrupt mitochondrial morphodynamics and ER‐mitochondria contact sites, promote mitochondrial fragmentation due to ΔΨ_m_ loss, and induce mitophagy through its viral nucleoprotein [65, 66, 67]; parainfluenza virus has also been associated with mitophagy induction [67]. Recent evidence underscores the pivotal role of mitochondria in SARS‐CoV‐2 pathogenesis [68]. Yeung‐Luk and colleagues demonstrated that interaction between the coronavirus spike protein and respiratory epithelial cells impairs mitochondrial metabolic activity via downregulation of NAD^+^‐dependent protein deacetylase 3 (SIRT3) and translocase of the outer mitochondrial membrane 22 (TOMM22) [69]. Another study showed that SARS‐CoV‐2 can directly impair Complex I of the ETC and suppress ATP synthase expression [70]. Collectively, these findings highlight mitochondria as potential targets of CRS‐related respiratory viruses.

#### 5.1.5 Allergens

Allergens are environmental triggers with a relevant role in the pathogenesis of allergic rhinitis, CRS, and asthma. These factors can be divided into indoor aeroallergens (including house dust mite, pet dander, and cockroach) and outdoor allergens (such as pollen grains, subpollen particles, and mold spores) [71]. A retrospective study found that CRS patients with house dust mite allergy were significantly younger at the time of surgery compared to nonallergic patients, suggesting that allergic inflammation may accelerate CRS progression [72]. Another study reported that house dust mite allergy may increase the severity of CRS in preschool and early school‐age children [73]. Allergic rhinitis, CRS, and asthma are now recognized as components of united airway disease (UAD), reflecting their shared morpho‐functional connection and overlapping pathogenic mechanisms, including epithelial barrier dysfunction, impaired MCC, dysregulated immune responses, and excessive tissue remodeling [74].

Experiments using asthma models have revealed a connection between mite‐induced airway barrier dysfunction and impaired mitochondrial biogenesis. In an in vitro experiment on alveolar epithelial cells, Aguilera‐Aguirre and colleagues showed that an exposure to pollen grains and subpollen particles could significantly impair the function of mitochondrial respiratory Complexes I and III. This study demonstrated that pollen‐derived NAD(P)H oxidases could impair ETC dysfunction by oxidizing ubiquinol‐cytochrome c reductase core protein I (UQCRC1) and II (UQCRC2) in Complex III and NADH dehydrogenase (ubiquinone) Fe‐S protein (NDUFS) 1 and NDUFS2 in Complex I [75]. Saito and colleagues demonstrated that exposure to house dust mites leads to downregulation of E‐cadherin in a human bronchial epithelial cell line (BEAS‐2B). The study further showed that mitochondrial biogenesis was significantly disrupted in these affected cells. This impairment was associated with the downregulation of PGC‐1α, a key upstream regulator of mitochondrial biogenesis, mediated through the PAR2/TLR4/PGC‐1α signaling pathway [76]. Bai and colleagues reported that house dust mites impair mitochondrial fusion and promote mitochondrial fission and mitophagy in BEAS‐2B cells by activating the DEK/ATAD3A/DRP1 signaling axis [77]. In addition, mitochondrial dysfunction has been implicated in the pathogenesis of mite‐induced allergic rhinitis. Zheng and colleagues demonstrated that exposure of nasal mucosa from patients with allergic rhinitis to house dust mites activates the NLRP3 inflammasome, leading to increased release of mtROS. The resulting oxidative stress contributes to mitochondrial dysfunction and the subsequent release of mtDNA, which, in turn, activates the STING pathway via the cGAS/STING (cyclic GMP‐AMP synthase/stimulator of interferon genes) pathway [78]. Given the shared pathogenic mechanisms between asthma and allergic rhinitis with CRS, it is reasonable to hypothesize that a similar disruption in mitochondrial biogenesis may occur in the context of CRS. Future studies are invited to examine this notion.

In a nutshell, various pathogenic factors implicated in CRS directly impair mitochondrial homeostasis in sinonasal epithelial cells, contributing to barrier dysfunction, oxidative stress, and persistent inflammation.

### 5.2 Epithelial Mitochondrial Damage in Exposure to CRS Risk Factors

This section outlines how established and potential CRS risk factors might affect the mitochondrial biogenesis.

#### 5.2.1 Tobacco

A large case‐control study denoted that tobacco use is a significant risk factor of CRS (adjusted odds ratio [OR] 1.42, 95% confidence interval [95% CI] 1.37–1.47) [79]. It has been demonstrated that tobacco can directly impair mitochondrial biogenesis by affecting mitochondrial respiration and morphology, impairing mitophagy, and forming mtDNA mutations and adducts [80].

#### 5.2.2 Alcohol

A study from China demonstrated that high alcohol consumption (> 7.5 drinks per week) is a significant risk factor of CRS [81]. Ethanol of alcoholic beverages can affect mitochondrial function through a myriad of mechanisms, including suppressing the main respiratory complexes involved in ETC, induction of oxidative stress, and mtDNA mutations [82].

#### 5.2.3 Obesity

A large, prospective study demonstrated that obesity (BMI ≥ 30) is a significant risk factor of CRS (OR 1.53, 95% CI 1.11–2.10) [83]. In support, a population‐based study from South Korea demonstrated a similar finding (OR 1.46, 95% CI 1.16–1.84) [84]. A recent meta‐analysis on more than 11 million patients with CRS from 20 countries corroborated this association [1]. In addition, a study from China on 555 patients with CRSwNP demonstrated that metabolic syndrome increases the risk of postoperative polyp recurrence [85]. It is well established that obesity significantly impairs mitochondrial function by inducing systemic low‐grade inflammation. Obesity‐induced reactive oxygen species (ROS) can damage mtDNA, membranes, and proteins [86]. In addition, obesity can impair mitochondrial quality control processes, including mitochondrial biogenesis, fission, fusion, and mitophagy, by affecting PGC‐1α [87]. Notably, mitochondrial dysfunction can itself increase the risk of developing obesity by lowering metabolic rates, thereby creating a vicious cycle that further exacerbates the condition [88].

#### 5.2.4 Aging

A large, population‐based study demonstrated that aging is a significant risk factor of CRSwNP (OR 1.03, 95% CI 1.02–1.04), with the highest prevalence in the seventh decade of life [84]. Mitochondrial dysfunction is a hallmark of aging process and age‐related diseases, like metabolic syndrome, neurodegenerative disease, and cardiovascular disorders. Several mechanisms have been proposed as the underlying mechanisms of aging‐related mitochondrial dysfunction, including accumulation of somatic mtDNA mutations, oxidative stress‐induced mitochondrial damage, altered mitochondrial dynamics, and mitophagy impairment [89].

#### 5.2.5 Air Pollution

A recent study on 376 participants demonstrated that high 12‐month exposure to particulate matter ≤ 2.5 µm in diameter (PM_2.5_) significantly increases the risk of CRSwNP. This study showed no association between PM_2.5_ exposure and risk of CRSsNP [90]. Mounting evidence points to mitochondria as the targets of PM_2.5_. The study by Leclercq and colleagues demonstrated that PM_2.5_ exposure affects mitochondrial function via oxidative and inflammatory pathways. Low‐dose, acute exposure activates nuclear factor erythroid 2‐p45 related factor 2 (NRF2), enhancing antioxidant gene expression, reducing ROS, and maintaining mitochondrial integrity, redox balance, and cellular homeostasis. In contrast, high‐dose or repeated exposure suppresses NRF2 signaling and activates nuclear factor kappa‐light‐chain‐enhancer of activated B cells (NF‐κB), resulting in elevated ROS, cytokine secretion, and mitochondrial dysfunction. This forms a vicious cycle of oxidative stress, impaired mitochondrial function, and inflammation, ultimately leading to cellular injury [91].

#### 5.2.6 Nonsteroidal Anti‐Inflammatory Drug–Exacerbated Respiratory Disease

Nonsteroidal anti‐inflammatory drug–exacerbated respiratory disease (N–ERD) is a well‐recognized risk factor for nasal polyposis. N–ERD affects approximately 0.3%–0.9% of the general population [92], and is characterized by a tetrad of asthma, CRSwNP, hypersensitivity to aspirin/NSAIDs, and an underlying eosinophilic Type‐2 inflammatory profile [93]. The evidence linking mitochondrial dysfunction to N–ERD pathogenesis remains nuanced.

Although no study has directly evaluated mitochondrial biogenesis in N–ERD, several observations collectively suggest a potential connection. In 2021, Haimerl and colleagues reported elevated levels of acylcarnitine in alveolar monocyte‐derived macrophages from patients with N–ERD [94]. Acylcarnitine is a key intermediate generated during fatty‐acid oxidation (FAO), reflecting the transport of fatty acyl groups into mitochondria (Figure 3B). FAO (AKA β‐oxidation) occurs primarily within mitochondria and depends on the conversion of fatty acids to acylcarnitine to enable their translocation across the mitochondrial membrane, where β‐oxidation takes place [95]. An increase in acylcarnitine may therefore indicate impaired mitochondrial FAO, leading to their intracellular accumulation. Such impairment may arise from several interconnected mechanisms: (1) dysfunction of the ETC, which slows β‐oxidation by preventing NADH and FADH_2_ utilization; (2) oxidative damage to key FAO enzymes; and (3) reduced TCA‐cycle flux, which creates metabolic “back‐pressure” limiting acetyl‐CoA entry and promoting incomplete FAO (Figure 3). Because myeloid cells, including macrophages and monocytes, rely heavily on FAO for their metabolic needs [96], elevated acylcarnitine levels in alveolar monocyte‐derived macrophages—as observed by Haimerl and colleagues—may reflect underlying mitochondrial dysfunction.

#### 5.2.7 Dietary Inflammatory Index

The role of diet in CRS is still emerging, but growing evidence suggests that dietary patterns may influence disease onset and symptom severity. Several observational studies have reported associations between specific dietary components and CRS outcomes: high intake of saturated fats and calories has been linked to and increased risk of CRS [97], while lower fruit consumption appears more common among individuals with CRS [98]. Taken together, these findings can be viewed through the framework of the Dietary Inflammatory Index (DII), in which diets with a high inflammatory potential (e.g., processed foods, saturated fat, sugar) may contribute to CRS exacerbation, whereas dietary patterns characterized by low DII scores (e.g., diets rich in fruits, vegetables, and fiber) may help alleviate symptoms or reduce disease burden. This assumption was demonstrated in a population‐based study from South Korea [97]. A study from Spain demonstrated that adherence to the Mediterranean diet—a prototypical low‐DII dietary pattern—was associated with a significant reduction in CRS severity [99].

It has been demonstrated that different components of the Mediterranean diet can positively affect the mitochondrial metabolism. For example, whole grains can improve mitochondrial respiration and ΔΨ_m_, delphinidin in berries has anti‐inflammatory features and can preserve mtDNA and Complex IV activity, and resveratrol present in grapefruits and lycopene present in tomatoes have anti‐inflammatory effect preserving mitochondrial enzymes [100]. In addition, a recent experimental study on rats demonstrated that high‐fiber diet could significantly improve the mitochondrial numbers compared with the high‐fat high‐fructose diet [101]. On the other hand, high‐DII diets can negatively affect the mitochondrial biogenesis [102]. This negative effect might be mediated through gut microbiome dysregulation and inflammation‐induced oxidative damage to mitochondrial structures [103].

#### 5.2.8 Abrupt Climate Change

Current evidence regarding the association between climate indices, such as temperature and humidity, and the development or exacerbation of CRS remains nuanced. A large study involving 4752 patients with CRS demonstrated that short‐term exposure to extreme heat significantly increased the odds of CRS symptom exacerbation (OR 1.11, 95% CI 1.03–1.19), with an observed lag period of 14 days. The authors suggested that elevated ambient temperature, through its effect on increasing ambient PM_2__.5_ concentrations, may contribute to the worsening of CRS symptoms [104].

In addition to this mechanism, the detrimental effects of abrupt heat exposure on mitochondrial biogenesis may further explain this association. Acute heat stress has been shown to reduce mitochondrial respiratory capacity, ATP synthesis, and mitochondrial gene expression, while simultaneously increasing mtROS production and mitophagy. In contrast, chronic or sustained heat exposure appears to have an adaptive effect, enhancing mitochondrial biogenesis by upregulating upstream regulatory pathways, including AMP‐activated protein kinase (AMPK), PGC‐1α, NRF2, and nuclear respiratory factor 1 (NRF1) [105]. These findings provide a plausible mechanistic explanation for how short‐term heat exposure may exacerbate CRS symptoms.

#### 5.2.9 Sleep Disturbance

Sleep disturbance is a common symptom in patients with CRS [106]. But can poor sleep quality actually increase the risk of developing CRS? A large population‐based cohort study sought to answer this question by following 5145 individuals who reported no nasal symptoms at baseline. After a 10‐year follow‐up, 141 participants (2.7%) had developed CRS. All sleep‐related problems, including difficulties initiating sleep, difficulties maintaining sleep, early morning awakening (EMA), snoring, and excessive daytime sleepiness (EDS), were more prevalent at baseline among individuals who later developed CRS. At follow‐up, these sleep problems and EDS had increased in both groups, but the rise was substantially greater among those who developed CRS. Notably, EMA and EDS emerged as independent risk factors for CRS in adjusted models (OR = 3.06, 95% CI 1.83–5.11; and OR = 1.79, 95% CI 1.19–2.68, respectively). Moreover, having multiple insomnia symptoms at baseline was strongly associated with CRS incidence at follow‐up: individuals reporting two symptoms had more than twice the risk (OR = 2.37, 95% CI 1.24–4.51), while those with three symptoms had a five‐fold increased risk (OR = 5.00, 95% CI 1.93–12.99) [107]. These findings underscore a bidirectional relationship between sleep disturbance and CRS, forming a vicious cycle in which poor sleep quality increases the risk of developing CRS, while CRS itself further disrupts sleep, perpetuating symptom burden.

The association between sleep quality and mitochondrial health has been demonstrated in several studies. A population‐based study from South Korea reported that poor sleep quality, prolonged sleep latency, and shift work were significantly associated with lower mtDNA copy number, a marker of mitochondrial biogenesis [108]. This relationship may be explained, at least in part, by differences in serum melatonin levels, which are markedly higher in individuals with good sleep quality [109]. Melatonin plays a crucial role in regulating mitochondrial function by promoting mitochondrial fusion [110], supporting ETC activity [111], and protecting mitochondrial integrity through its intrinsic antioxidant properties [111]. In essence, mitochondria rely on effective sleep and the associated melatonin signaling to maintain their longevity and optimal function.

In summary, various CRS risk factors disrupt mitochondrial homeostasis in sinonasal epithelial cells, exacerbating inflammation, oxidative stress, and barrier dysfunction.

### 5.3 Eosinophil's Mitochondrial Activation in the Context of CRS

Emerging evidence demonstrates that eosinophils undergo pronounced mitochondrial activation in the context of allergic asthma. Using an allergen‐induced murine model, Bonjour and colleagues showed that bone marrow‐derived eosinophils from asthmatic mice exhibit increased mitochondrial number and volume, accompanied by striking cristae remodeling: a shift from exclusively lamellar structures in naïve eosinophils to a mixed lamellar–tubular architecture in inflammatory eosinophils. Mature eosinophils displayed even greater mixed‐cristae morphology than their immature counterparts. These structural adaptations were associated with enhanced OXPHOS capacity, enabling the cells to meet the elevated ATP and biosynthetic requirements during type‐2 inflammation [112]. Complementary human studies have confirmed that IL‐5 and GM‐CSF, key cytokines in allergic asthma, drive both glycolysis and OXPHOS in blood eosinophils via STAT5‐ and PI3K/Akt‐dependent pathways. In addition, NOX‐derived ROS can promote OXPHOS and TCA in eosinophils [113]. These findings further underscore mitochondrial activation as a hallmark of eosinophil effector function in allergic airway disease. In addition, IL‐5 can improve eosinophils' survival by inhibiting the translocation of Bax protein, a proapoptotic component, into mitochondria [114].

Taken together, these data establish mitochondrial activation, encompassing increased biogenesis, cristae remodeling, enhanced OXPHOS, and protection against apoptosis, as a central metabolic hallmark of eosinophil effector responses in allergic asthma. Given the extensive overlap in pathogenic mechanisms between asthma and CRS, it is reasonable to expect similarly enhanced mitochondrial biogenesis in eosinophils within CRS.

### 5.4 Inflammation, Oxidative Stress, and Mitochondrial Dysfunction: A Vicious Cycle of CRS

As noted above, the mitochondria of epithelial cells can be directly affected by various CRS triggers (see Section 5.1). Additionally, exposure to these factors can directly induce oxidative stress in nasal polyp tissue. For instance, Jiang and colleagues demonstrated that LPS can trigger oxidative stress in nasal polyp tissue by activating NADPH oxidase 2 (NOX2) [115]. The primary pathogenic mechanism of CRS triggers likely involves the induction of progressive, self‐perpetuating inflammation in the sinonasal tissue, initiating a vicious cycle that ultimately leads to epithelial mitochondrial dysfunction. This section highlights the interplay between the inflammatory response to CRS‐related pathogenic factors, oxidative stress, and mitochondrial dysfunction, together generating a vicious cycle central to CRS pathogenesis (Figure 5):

**Figure 5:**
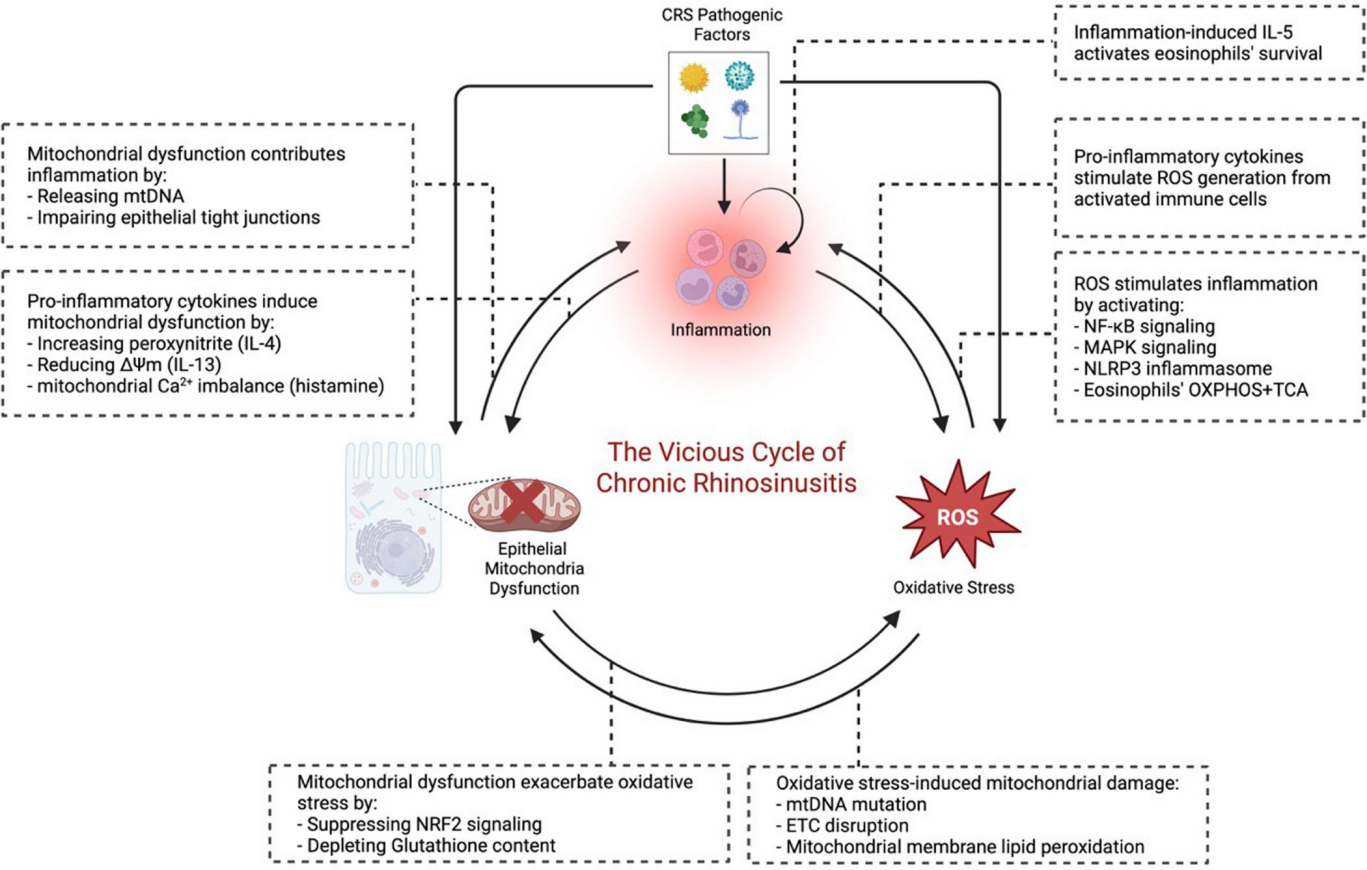
The vicious cycle between pathogenic factors‐induced inflammation, oxidative stress, and mitochondrial dysfunction in the context of chronic rhinosinusitis. ΔΨ_m_, mitochondrial membrane potential; ETC, electron transport chain; IL‐4, interleukin‐4; IL‐13, interleukin‐13; MAPK, mitogen‐activated protein kinase; mtDNA, mitochondrial DNA; NF‐κB, nuclear factor kappa‐light‐chain‐enhancer of activated B cells; NLRP3, nucleotide‐binding oligomerization domain‐like receptor family pyrin domain containing 3; NRF2, nuclear factor erythroid 2–related factor 2; ROS, reactive oxygen species. Created in BioRender. Taghizadeh‐Hesary (2026) https://BioRender.com/k5f4t1f.

#### 5.4.1 Progressive Inflammation Initiates Oxidative Stress

In CRS, exposure to triggers activates the innate immune system, leading to a self‐perpetuating inflammatory response. The activated immune cells secrete pro‐inflammatory cytokines, which in turn stimulate the production of ROS as part of the host defense mechanism. Jones and colleagues demonstrated that IL‐5 can directly enhance ROS generation by activating the eosinophils' OXPHOS in a STAT5/PI3K/Akt‐dependent manner [113]. While ROS are essential in microbial killing, excessive ROS generation leads to oxidative stress, damaging surrounding host tissues [4]. In this inflamed microenvironment, epithelial and endothelial cells are subjected to continuous ROS exposure, which overwhelms their intrinsic antioxidant defenses, setting the stage for their mitochondrial injury.

#### 5.4.2 Oxidative Stress Impairs Mitochondrial Function

Mitochondria are both sources and targets of ROS. Under homeostatic conditions, ROS produced during OXPHOS are detoxified by mitochondrial antioxidants such as SOD2 and glutathione peroxidase (GPx) [116]. However, sustained oxidative stress in CRS can surpass this detoxification capacity, leading to cumulative mitochondrial damage [117]. Key consequences include: (1) mtDNA mutations: These disrupt the production of proteins essential for ETC function and ROS management, further weakening the mitochondrial resilience [118]; (2) Disruption of ETC: ROS‐induced damaged Complexes I and III exhibit electron leak, favoring the formation of superoxide anions (O2•^−^) (see Section 5.1.1); and (3) Lipid peroxidation: generated ROS can attack mitochondrial membranes, compromising the function of embedded proteins, including those of the ETC. This disruption further impairs membrane integrity and mitochondrial function by promoting additional ROS generation through electron leakage [119]. These events create a feed‐forward loop in which damaged mitochondria produce increasing amounts of ROS, thereby intensifying cellular stress.

#### 5.4.3 Mitochondrial Dysfunction Amplifies Oxidative Stress

Secondary to oxidative stress mitochondrial antioxidant defenses are compromised by: (1) Suppression of NRF2 signaling: NRF2, a transcription factor responsible for upregulating antioxidant nuclear genes, becomes dysregulated in chronic stress settings. Leclercq and colleagues demonstrated that repeated exposure to PM_2.5_ suppresses NRF2 while activating NF‐κB, tipping the balance toward oxidative and inflammatory responses [91]; (2) Antioxidant depletion: Prolonged ROS generation can deplete glutathione, thereby impairing essential detoxification pathways. Moreover, glutathione depletion hinders the regeneration of key antioxidants such as vitamin E and vitamin C [120]; and (3) Impaired glutathione regeneration due to ETC dysfunction: The regeneration of reduced glutathione (GSH) from its oxidized form (GSSG) is catalyzed by glutathione reductase, a process that requires nicotinamide adenine dinucleotide phosphate (NADPH) as a cofactor [120]. While NADPH is primarily produced through cytosolic pathways such as the pentose phosphate pathway and the malic enzyme, mitochondrial function plays a critical supporting role in sustaining cellular NADPH levels particularly through mitochondrial isocitrate dehydrogenase activity [121].

#### 5.4.4 Oxidative Stress Feeds Back to Intensify Inflammation

Excess mtROS can leak into the cytosol, triggering oxidative stress and functioning as secondary messengers that activate redox‐sensitive signaling cascades. These include: (1) NF‐κB, which drives the transcription of pro‐inflammatory cytokines such as IL‐6, IL‐8, and eotaxin [122]; (2) mitogen‐activated protein kinase (MAPK) pathways, including p38, which amplify cellular stress responses and promote the production of cytokines like IL‐1, IL‐6, and IL‐8 [123]; and (3) the NLRP3 inflammasome (nucleotide‐binding oligomerization domain‐like receptor family pyrin domain containing 3), which facilitates the secretion of IL‐1β and IL‐18, further enhancing immune cell recruitment by stimulating epithelial cells to release alarmins such as IL‐25, IL‐33, and TSLP [115]. Clinical evidence has also demonstrated that this ROS‐driven signaling cascade establishes a pro‐inflammatory feedback loop. Hu et al. demonstrated that oxidative stress contributes to enhanced resistance to steroids in patients with CRSwNP [124].

#### 5.4.5 Mitochondrial Damage Exacerbates Inflammation

Beyond their role in oxidative stress, damaged mitochondria actively contribute to the amplification of inflammation in CRS through multiple mechanisms: (1) One such mechanism involves the release of mtDNA, which acts as a potent danger‐associated molecular pattern (DAMP). Zheng and colleagues demonstrated that the mitochondrial enzyme cytidine/uridine monophosphate kinase 2 (CMPK2) is upregulated in the nasal mucosa of patients with allergic rhinitis and in house dust mite‐challenged murine models. CMPK2 promotes the release of oxidized mtDNA, which subsequently activates the NLRP3 inflammasome via the cGAS/STING signaling axis [78]; (2) In addition to inflammasome activation, mitochondrial dysfunction exacerbates local inflammation by disrupting tight junction (TJ) integrity within the nasal epithelium (see Section 6.1). The resulting epithelial barrier dysfunction facilitates increased exposure to airborne antigens, microbes, and allergens, leading to sustained immune cell recruitment and persistent inflammatory signaling [4].

#### 5.4.6 Inflammation Products Affect Epithelial and Eosinophils' Mitochondrial Function

Inflammatory mediators, particularly cytokines, play a pivotal role in modulating mitochondrial function. In the context of chronic inflammation, such as in CRS, sustained exposure to these cytokines can disrupt epithelial cells' mitochondrial dynamics, bioenergetics, and redox balance, contributing to epithelial dysfunction and disease progression. In the following, the impacts of different cytokines on the epithelial cells' mitochondrial homeostasis are outlined.

IL‐4 stimulates the proteolytic degradation of dimethylarginine dimethylaminohydrolase 2 (DDAH2), leading to the accumulation of asymmetric dimethylarginine (ADMA) [125]. Elevated levels of ADMA disrupt endothelial nitric oxide synthase (eNOS) function, causing it to become uncoupled [125]. This uncoupling results in increased production of ROS and peroxynitrite, both of which contribute to mitochondrial dysfunction [126]. IL‐13: Zhu and colleagues demonstrated that IL‐13 can directly inhibit mitochondrial metabolism, marked by reduction in mitochondrial ΔΨ_m_, ATP levels, and SOD2 expression [127]. In addition to these findings, other cytokines can induce mitochondrial dysfunction in other tissues; for example, IL‐1 in mesothelial cells [128], TNF‐ α in renal tubular epithelial cells and neurons [129, 130], and TSLP in human monocytes [131].

Furthermore, inflammation‐induced histamine release can impair mitochondrial function by promoting inositol 1,4,5‐trisphosphate (IP_3_)‐mediated calcium release from the ER [51]. This increases mitochondrial calcium loading, which disrupts mitochondrial function through several mechanisms: (1) opening of the mPTP, leading to mitochondrial swelling and loss of ΔΨ_m_ [132, 133]; (2) overstimulation of the ETC, resulting in excessive mtROS generation due to uncoupling [134]; and (3) disruption of mitochondrial dynamics via enhanced DRP1‐mediated mitochondrial fission [135].

As noted in Section 3.1, JAK/STAT signaling axis plays a crucial role in cytokine‐induced inflammation. Emerging evidence has demonstrated that JAK‐induced STAT3 phosphorylation can impair mitochondrial function. Liu and colleagues demonstrated that JAK2/STAT3 activation could impair mitochondrial homeostasis, leading to oxidative stress‐induced necroptosis of renal cells [136]. Another experiment on keratinocytes demonstrated that STAT3 ablation could improve mitochondrial gene expression. This study demonstrated that this effect can be mediated by increase in the expression of PGC‐1α and NRF‐1–two mitochondrial upregulators [137]. STAT3 phosphorylation occurs in two principal forms: tyrosine phosphorylation (Tyr705), which primarily drives its nuclear transcriptional activity, and serine phosphorylation (Ser727), which modulates mitochondrial function and bioenergetics [138]. Notably, in the context of CRSwNP, STAT3 activation predominantly involves Tyr705 phosphorylation [139], suggesting that the JAK/STAT axis mainly exerts its pathogenic effects through transcriptional reprogramming rather than direct mitochondrial signaling [140]. Therefore, it can be concluded that aberrant JAK/STAT activation in CRS indirectly contributes to mitochondrial dysfunction, likely through sustained inflammatory signaling and metabolic dysregulation.

In contrast to the epithelial cells, inflammation‐induced IL‐5 can improve eosinophils' mitochondria in terms of integrity, structure, and function (Section 5.3) [112, 113]. Thus, within the inflamed sinonasal microenvironment of CRS, epithelial and endothelial cell mitochondria undergo progressive dysfunction due to overwhelming oxidative stress, whereas eosinophil mitochondria exhibit enhanced integrity, structural remodeling, and functional activation driven by IL‐5, highlighting two strikingly divergent mitochondrial fates in the same type‐2 inflammatory milieu.

#### 5.4.7 Mitochondrial Versus Plasma Membrane‐Derived ROS

The sinonasal epithelium harbors multiple ROS‐generating systems, including plasma membrane‐localized NADPH oxidases (primarily NOX2) and mitochondrial ETC complexes. NOX2 has been directly implicated in CRSwNP pathogenesis, where it drives ROS accumulation and NLRP3 inflammasome activation [115], likely serving as an initiating signal within the inflammatory cascade.

However, the pathological significance of mtROS derives from its capacity to sustain and amplify the inflammatory cycle. Unlike NOX2, which can be downregulated upon resolution of the triggering stimulus, mtROS originates from structurally and functionally damaged mitochondria [7]. Once mitochondrial injury occurs, a self‐perpetuating loop is established: damaged mitochondria produce excessive mtROS, which further impairs mitochondrial function, which in turn produces more mtROS. This feed‐forward mechanism is absent in NOX2‐mediated ROS generation. Moreover, mitochondrial damage releases mtDNA, which acts as a DAMP to activate cGAS/STING and NLRP3 signaling, adding another layer of inflammation amplification that NOX2‐derived ROS cannot directly initiate.

## 6 Histopathological Changes of CRS From the Lens of Mitochondria

This section outlines how mitochondrial dysfunction takes part in the common histopathological changes of sinonasal epithelium in the context of CRS (Figure 6).

**Figure 6:**
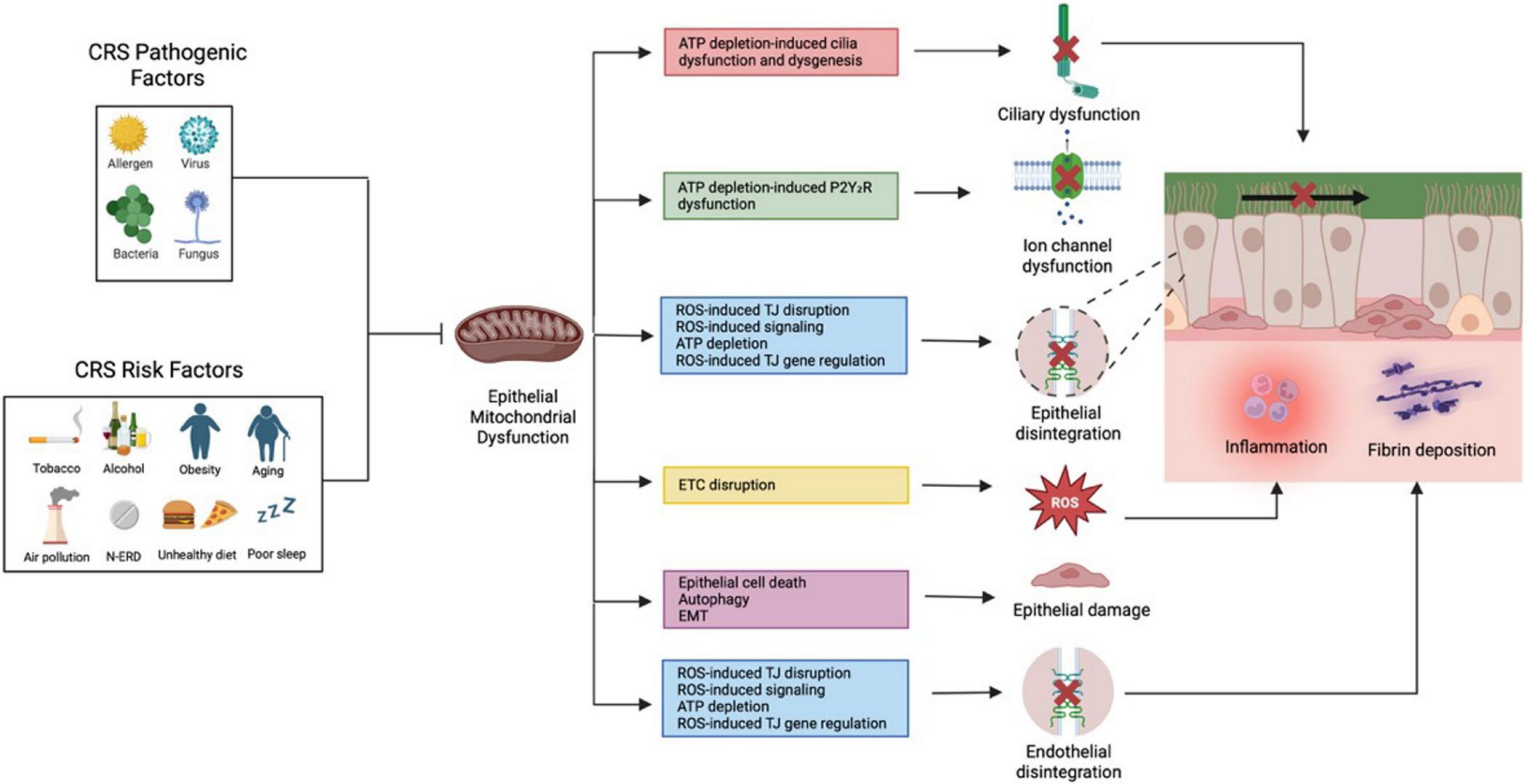
Mechanisms by which mitochondrial dysfunction contributes to histopathological changes in chronic rhinosinusitis. Key pathways are highlighted in colored boxes. ATP, adenosine triphosphate; CRS, chronic rhinosinusitis; EMT, epithelial–mesenchymal transition; ETC, electron transport chain; N–ERD, nonsteroidal anti‐inflammatory drug–exacerbated respiratory disease; P2Y_2_R, P2Y_2_ receptor; ROS, reactive oxygen species; TJ, tight junction. Created in BioRender. Taghizadeh‐Hesary (2026) https://BioRender.com/03dfofl.

### 6.1 Epithelial Disintegration

Tight junctions (TJs) are key components of epithelial and endothelial barrier integrity. They regulate paracellular permeability, prevent the translocation of pathogens and allergens, and maintain mucosal homeostasis in the upper airway. Tight junction proteins such as ZO‐1, claudins, occludin, junctional adhesion molecules, and E‐cadherin are essential for maintaining the integrity of the sinonasal epithelial as well as endothelial barriers [141]. This section outlines the mechanism by which mitochondrial dysfunction disrupts TJ proteins expression and function.

#### 6.1.1 ROS‐Induced Disruption of Tight Junction Proteins

One of the principal mechanisms by which oxidative stress compromises epithelial and endothelial barrier integrity is through ROS‐induced destabilization of TJ proteins. A substantial body of evidence has demonstrated that this represents a major pathway underlying oxidative stress–mediated barrier dysfunction. In this process, excessive ROS modify and destabilize TJ proteins through post‐translational modifications, including phosphorylation, thiol oxidation, carbonylation, and nitration, ultimately leading to disruption of intercellular junctions and increased barrier permeability [45]. Kim and colleagues demonstrated that rhinovirus impairs nasal epithelial junctional proteins through ROS‐mediated inactivation of protein phosphatases [142]. As noted in Section 5.1, rhinovirus can also damage epithelial cells by increasing mtROS production through disruption of the mitochondrial ETC [62]. Therefore, rhinovirus‐induced TJ dysfunction can be regarded as a mitochondria‐dependent process.

#### 6.1.2 Oxidative‐Induced Signaling

In addition to post‐translational modification of TJ proteins, oxidative stress can also compromise epithelial barrier function through the activation of ROS‐sensitive intracellular signaling pathways. In this mechanism, ROS act as secondary messengers, triggering cascades that regulate TJ protein expression and stability. For instance, a study on human bronchial epithelial cells demonstrated that elevated intracellular ROS levels promote ZO‐1 and claudin‐2 degradation via activation of the TRPM2–PLCγ1–PKCα signaling pathway [143]. Similarly, research on human nasal epithelial cells showed that PM_2.5_ exposure downregulates TJ proteins, including ZO‐1, claudins, occludin, and E‐cadherin, through an Akt‐dependent mechanism [123]. As noted in Section 5.2.5, high‐dose or chronic exposure to PM_2.5_ can also lead to mitochondrial dysfunction and excessive mtROS generation [91]. Collectively, these findings indicate that an increase in cellular ROS, including mtROS, can disrupt epithelial integrity at multiple regulatory levels, including translational, post‐translational, and degradative processes.

#### 6.1.3 ATP Depletion

A study on oral epithelium demonstrated that epithelial cells react to invading bacteria by enhancing the expression of TJ proteins. Guo et al. showed that in response to *Porphyromonas gingivalis* (*P. gingivalis*), epithelial cells upregulate various TJ proteins in an ATP‐dependent manner [144]. Mitochondria are the primary source of ATP in human cells [145]; therefore, well‐functioning mitochondria are essential for maintaining intact TJ expression in response to pathogens. Although this study was conducted on oral epithelial cells, the structural and functional similarities between oral and nasal epithelial/endothelial barriers suggest that a comparable mechanism may be present in the sinonasal mucosa. However, further research is needed to directly confirm this relationship in nasal tissues.

#### 6.1.4 ROS‐Induced Gene Regulation

In addition to the previously described mechanisms, mtROS can directly regulate the expression of genes involved in TJ integrity. Kim and colleagues demonstrated that exposure of epithelial cells to *Aspergillus* proteases can disrupt the mitochondrial ETC, leading to increased mtROS production. This elevation in mtROS subsequently impairs the expression of ZO‐1, claudin‐1, occludin, and E‐cadherin [56]. In another study, Zhao and colleagues reported that exposure of nasal epithelial cells to PM_2.5_ downregulates the expression of TJ proteins, including ZO‐1, claudins, and occludin. This detrimental effect was alleviated by treatment with *N*‐acetyl‐l‐cysteine, a ROS scavenger [146]. This finding indicates that PM_2.5_‐induced suppression of TJ proteins is an ROS‐dependent process. As noted above, high‐dose or repeated exposure to PM_2.5_ can increase mtROS release [91]. Therefore, the deleterious effects of PM_2.5_ on nasal epithelial barrier integrity are likely mediated through mitochondria‐dependent oxidative mechanisms.

These findings indicate that mitochondrial dysfunction contributes to both epithelial and endothelial barrier disruption, acting through multiple interrelated mechanisms.

### 6.2 Ciliary Dysfunction

MCC is a vital component of sinonasal mucosal defense, protecting the airways against inhaled particulates, pathogens, and irritants. As noted in Section 3.1, ciliary dysfunction is a key feature in the pathogenesis of CRS [4]. The cilia on the sinonasal epithelium exhibit the characteristic axonemal structure, consisting of a “9 + 2” arrangement of microtubules. These microtubules are surrounded by outer and inner dynein arms, which form the motor complexes responsible for generating coordinated, rhythmic bending motions. This process is ATP‐dependent, relying on sufficient mitochondrial energy production [147, 148]. In support of this, evidence indicates that in human epithelial cells, mitochondria localize to the apical surface adjacent to the cilia, enhancing the efficiency of ATP delivery [149]. In addition, Lo and colleagues demonstrated that ATP is essential for stabilizing the protein structure of the transition zone of cilia, as the foundation of primary cilia structure, and promotes the assembly of its protein structure [150].

Consequently, mitochondrial dysfunction induced by CRS‐associated triggers or environmental risk factors can impair both ciliary formation and motility, leading to defective MCC and persistent inflammation.

### 6.3 Airway Surface Liquid

ASL volume is preserved through the coordinated activity of several ion transport channels and regulatory pathways on airway epithelial cells. Cystic fibrosis transmembrane conductance regulator (CFTR), a cyclic AMP–regulated Cl^−^ channel, drives chloride and bicarbonate secretion, pulling water into the lumen to hydrate the ASL, whereas the epithelial sodium channel (ENaC) absorbs sodium and reduces ASL volume [151]. The P2Y_2_ receptor (P2Y_2_R) further promotes ASL hydration by stimulating Ca^2+^‐dependent chloride secretion and concurrently inhibiting ENaC activity. Proper ASL homeostasis depends on the coordinated regulation of these opposing pathways, with both CFTR and P2Y_2_R helping to limit excessive ENaC‐mediated absorption. Similar to ciliary motion, preserving ASL volume is an ATP‐dependent process, as epithelial cells release extracellular ATP into ASL that activates P2Y_2_R, thereby enhancing chloride secretion and transiently expanding ASL volume [152]. Therefore, mitochondrial homeostasis plays a crucial role in preserving ASL volume by providing enough ATP molecules.

### 6.4 Epithelial Cells Changes

In the context of CRS, sinonasal epithelial cells can undergo various changes, including different forms of cell death, disrupted or dysregulated autophagy, and activation of epithelial–mesenchymal transition (EMT) [153, 154, 155].

#### 6.4.1 Cell Death Mechanisms

It has been demonstrated that epithelial cells undergo various forms of regulated cellular death during the CRS pathogenesis. This includes apoptosis, necroptosis, ferroptosis, entotic cell death, NETosis, lysosome‐dependent cell death, immunogenic cell death, and anoikis [153]. Compelling evidence indicates that mitochondrial dysfunction is central to several epithelial cell death mechanisms in CRS. (1) Apoptosis: In CRSwNP, the pro‐apoptotic protein Bax is upregulated while the antiapoptotic protein BCL‐2 is downregulated [156]. Mitochondrial dysfunction amplifies this imbalance by promoting ROS‐dependent Bax activation and ROS‐mediated suppression of BCL‐2 expression [157, 158]; (2) Necroptosis: Necroptosis is activated in both eosinophilic and noneosinophilic CRSwNP [159], primarily through the TNF‐α/RIPK1/RIPK3/MLKL signaling axis [160]. Notably, MLKL can translocate to mitochondria, where it promotes mtDNA release via a microtubule‐dependent process; the released mtDNA subsequently triggers inflammation through activation of the cGAS–STING pathway [161]; (3) Ferroptosis: Ferroptosis is an iron‐dependent, nonapoptotic cell death pathway characterized by excessive lipid peroxidation [160]. CRS‐associated triggers can promote ferroptosis by disrupting ETC function and enhancing ROS accumulation (Section 5.1). Elevated ROS oxidize polyunsaturated phospholipids (PUFA‐PLs) into phospholipid hydroperoxides, driving ferroptotic death [160]. Together, these findings suggest that mitochondrial dysfunction‐induced oxidative stress represents a potential mechanism underlying multiple epithelial cell death pathways in CRS.

ROS accumulation can drive epithelial cells toward cell death. Proper mitochondrial metabolism helps counteract the detrimental effects of oxidative stress by supporting the regeneration of key redox‐buffering systems, including glutathione and thioredoxin [162]. In addition, Jain and colleagues demonstrated that epithelial cells can prevent excessive ROS generation through mitochondrial uncoupling proteins. In this mechanism, mitochondrial uncoupling protein can serve as a “safety valve,” releasing the accumulated intermitochondrial membrane H^+^ into mitochondrial matrix, without generating ATP molecules [149]. With this strategy, epithelial cells try to preserve their ROS levels at nontoxic levels at the expense of reducing mitochondrial ATP production.

#### 6.4.2 Autophagy

It has been demonstrated that autophagy processes are disrupted in CRSwNP. Wang and colleagues reported that both autophagy and mitophagy are reduced in the nasal tissue of patients with CRSwNP, likely due to activation of the Akt/mTOR pathway [163]. Consistently, another study showed that LC3, a key autophagy marker, is markedly decreased in CRSwNP, and that pharmacologic activation of autophagy effectively reduced COX‐2 expression, an indicator of inflammation, in nasal polyp–derived fibroblasts, whereas IL‐1β and TNF‐α did not influence autophagy levels [154]. These findings suggest that mechanisms beyond inflammatory cytokine signaling contribute to autophagy impairment in CRS. Mitochondrial biogenesis plays a pivotal role in regulating autophagy. Graef and Nunnari demonstrated that mitochondrial respiratory deficiency suppresses both autophagic flux and the expression of autophagy‐related genes [164]. Together, these observations indicate that mitochondrial dysfunction may represent an important upstream driver of autophagy disruption in CRS.

#### 6.4.3 Epithelial–Mesenchymal Transition

Emerging evidence highlights the importance of EMT in the pathogenesis of CRS. However, EMT appears to be more prominent in CRSwNP, while findings in CRSsNP remain inconsistent [155]. Mitochondria are the primary source of cellular ROS [165], and various CRS‐associated pathogenic factors can exacerbate mitochondrial ROS production by disrupting mitochondrial homeostasis (Section 5.1). Accumulated ROS have been shown to promote EMT in patients with CRSwNP [166]. Therefore, although no study has directly examined the role of mitochondrial biogenesis in CRS‐related EMT, current evidence supports its involvement indirectly through mitochondrial dysfunction–induced oxidative stress. Further studies are required to clarify this relationship and to determine whether restoring mitochondrial homeostasis can modulate EMT and potentially improve clinical outcomes in CRS.

### 6.5 Fibrin Deposition

Fibrin deposition is a critical step in nasal polyp formation in CRS. This process begins with endothelial cell dehiscence and leakage of plasma components into the nasal tissue, followed by activation of the coagulation cascade and subsequent fibrin accumulation within the sinonasal mucosa. The rate of fibrin deposition in CRS tissue is reported to be 10–20 times higher than in healthy sinonasal mucosa [4]. The accumulated fibrin forms a mesh‐like structure that traps water and contributes to the edematous nature of nasal polyps [167]. Imoto and colleagues showed that activation of the coagulation cascade, reflected by elevated levels of the coagulation markers TAFI and TATc, correlates with CRS severity [168]. As noted earlier, this sequence is initiated by endothelial disintegration secondary to TJ disruption. Therefore, the mechanisms outlined in Section 6.1, which describe how mitochondrial dysfunction can compromise TJ integrity, may also underlie endothelial breakdown and the subsequent fibrin deposition observed in CRS.

## 7 The Association Between CRS‐Related Genetic/Epigenetic Factors and Mitochondrial Homeostasis

### 7.1 Downregulated Genes: Cystic Fibrosis Transmembrane Conductance Regulator

CFTR is an epithelial cells' transmembrane channel, transferring Cl^−^ from intraepithelial cells' cytosol into ASL. CF is a genetic disease caused by mutations in the *CFTR* gene [169]. Emerging evidence has demonstrated that *CFTR* mutation makes epithelial cells mitochondria vulnerable to the detrimental effects of CRS‐related pathogenic factors. Rimessi and colleagues showed that defective CFTR expression increases mitochondrial vulnerability during *P. aeruginosa* infection. CF bronchial epithelial cells (IB3‐1 and CuFi‐1) exhibited exacerbated mitochondrial dysfunction, marked by ΔΨ_m_ reduction, elevated ROS, fragmented networks, and Ca^2+^ dysregulation, compared to CFTR‐intact S9 or NuLi cells. In that study, wild‐type CFTR expression preserved mitochondrial integrity, mitigating the pathogen‐induced decline in Ca^2+^ uptake, impaired ΔΨ_m_, and oxidative stress. This protection extended to limiting inflammasome activation and cytokine release. Additionally, silencing the mitochondrial calcium uniporter (MCU) in CF cells prevented flagellin‐induced mitochondrial perturbations and downstream NLRP3 signaling, underscoring the role of MCU as a target in *P. aeruginosa*‐induced inflammation [51]. These results suggest that epithelial cells' mitochondria are the targets in the CF‐associated *P. aeruginosa* stimuli, amplifying inflammation through dysregulation of Ca^2+^‐dependent mitochondrial signaling.

### 7.2 Upregulated Genes: ALOX15

Experimental evidence has shown that gene upregulation also contributes to the pathogenesis of CRSwNP. Through bioinformatic analysis, Yang and colleagues identified ALOX15 as a core gene involved in CRSwNP development and reported a positive correlation between ALOX15 expression and several inflammation‐related genes [170]. Beyond its inflammatory role, ALOX15 can also influence mitochondrial function. Pallast and colleagues demonstrated that ALOX15 directly damages mitochondria and disrupts mitochondrial membrane potential, leading to the release of cytochrome c. Their study further showed that mitochondrial interaction is a major source of ALOX15‐induced oxidative stress [171].

Together, these findings highlight how genetic alterations associated with CRSwNP pathogenesis can contribute to mitochondrial dysfunction.

## 8 Therapeutic Implications: Targeting Mitochondrial Dysfunction and Oxidative Stress

Given the central role of mitochondrial dysfunction and oxidative stress in the CRS “vicious cycle” (Figure 5), therapeutic strategies targeting these pathways warrant investigation. Several agents have shown promise in preclinical models.

### 8.1 Mitochondria‐Targeted Antioxidants

MitoQ, NecroX‐5, and MitoTEMPO have demonstrated efficacy in reducing mtROS in CRS‐relevant experimental systems. MitoQ and NecroX‐5 alleviated LPS/H_2_O_2_‐induced mtROS production in human nasal tissue explants and rescued mtDNA depletion [11, 12]. MitoTEMPO prevented SEB‐induced mitochondrial damage and oxidative stress in primary human nasal epithelial cells [10]. These findings suggest that mitochondria‐specific antioxidants may protect sinonasal epithelium from oxidative injury.

### 8.2 Nrf2 Pathway Activators

As noted in Section 5.4.3, mitochondrial dysfunction exacerbates oxidative stress in epithelial cells by suppressing NRF2 signaling. Dietary supplements can modify this axis. Sulforaphane (SFN), a dietary isothiocyanate, activates Nrf2‐dependent antioxidant responses. In a murine model of allergic rhinosinusitis, SFN reduced eosinophil accumulation and eotaxin‐1 levels while stabilizing epithelial barrier function [172]. A pilot human study demonstrated that SFN‐rich broccoli sprout homogenate enhanced secretory leukocyte protease inhibitor secretion in nasal mucosa within 48 h [173].

### 8.3 Emerging Targets

APE1 is a multifunctional enzyme whose N‐terminal domain induces mitochondrial damage through oxidative stress. In a mouse CRS model, the specific APE1 inhibitor C10 reduced type 2 inflammation and mitochondrial oxidative stress [174]. However, these findings remain preclinical, and clinical trials in CRS patients are needed to establish safety and efficacy.

### 8.4 Challenges and Future Directions

Despite promising preclinical results, clinical translation of mitochondria‐targeted therapies faces several obstacles. The optimal delivery route for sinonasal application remains undefined; intranasal administration may improve local concentration but requires formulation optimization. Patient selection based on oxidative stress biomarkers could identify those most likely to benefit. Combination strategies targeting both mtROS production and NRF2‐mediated antioxidant capacity may yield synergistic effects. Finally, randomized controlled trials with standardized clinical outcomes are needed to establish safety and efficacy in CRS patients.

## 9 Conclusions

This narrative review is the first to comprehensively describe how mitochondria dysregulation contributes to the pathogenesis of CRS. By synthesizing evidence from diverse experimental studies, we evaluated mitochondrial involvement across key aspects of CRS physiopathology, including mitochondrial damage induced by pathogens and lifestyle and environmental risk factors, the reciprocal interactions between inflammation, oxidative stress, and mitochondrial dysfunction, and the resulting histopathological changes in CRS tissue. Collectively, these findings highlight a self‐perpetuating vicious cycle in which epithelial cells' mitochondrial dysfunction increases ROS production and inflammatory signaling, while chronic inflammation and oxidative stress further impair mitochondrial integrity. Disrupting this cycle appears essential for reducing disease progression. On the other hand, current evidence favors mitochondrial enhancement in the eosinophils, as the leading immune components in T2‐type CRS.

Traditional CRS medical therapies such as corticosteroids and targeted monoclonal antibodies may partially act by attenuating the vicious cycle between inflammation and epithelial damage. In this study, we highlighted that this vicious cycle has another component, the epithelial cells' mitochondria. As such, beyond the aforementioned approaches, epithelial cells' mitochondria‐enhancing strategies, including metabolic supplements, mitochondria‐targeted antioxidants, nutritional interventions, and natural compounds, represent promising avenues for restoring mitochondrial homeostasis and improving epithelial function. This study also highlighted that mitochondrial biogenesis and function are enhanced in eosinophils. Therefore, mitochondria‐targeting strategies can serve as a potential approach to alleviate T2‐type inflammation in CRS.

While this review underscores the strong interplay between mitochondrial dysregulation and CRS pathogenesis, current evidence is insufficient to fully explain nasal polyp formation solely through mitochondrial metabolic pathways. Future studies should clarify the mitochondrial mechanisms distinguishing CRSwNP from CRSsNP and assess whether targeting mitochondrial health can provide therapeutic or preventive benefit in CRS.

## Author Contributions

**Babak Ghalehbaghi:** conceptualization; writing – review and editing. **Morteza Fallahpour:** writing – review and editing. **Mohammad Nabavi:** supervision, writing – review and editing. **Maryam Jalessi:** writing – review and editing. **Hadi Ghanbari:** visualization. **Nasibeh Khayer:** writing – review and editing. **Farzad Taghizadeh‐Hesary:** conceptualization, methodology, software, investigation, writing – original draft, writing – review and editing.

## Funding

The authors have nothing to report.

## Ethics Statement

The authors have nothing to report.

## Consent

The authors have nothing to report.

## Conflicts of Interest

The authors declare no conflicts of interest.

## Data Availability

The authors have nothing to report.
